# Deep mutational scanning of the RNase III-like domain in *Trypanosoma brucei* RNA editing protein KREPB4

**DOI:** 10.3389/fcimb.2024.1381155

**Published:** 2024-04-08

**Authors:** Suzanne M. McDermott, Vy Pham, Brian Oliver, Jason Carnes, D. Noah Sather, Kenneth D. Stuart

**Affiliations:** ^1^ Center for Global Infectious Disease Research, Seattle Children’s Research Institute, Seattle, WA, United States; ^2^ Department of Pediatrics, University of Washington School of Medicine, Seattle, WA, United States

**Keywords:** deep mutational scanning, *Trypanosoma brucei*, RNase III, parallel mutagenesis, RNA editing, RNA editing catalytic complex

## Abstract

Kinetoplastid pathogens including *Trypanosoma brucei*, *T. cruzi*, and *Leishmania* species, are early diverged, eukaryotic, unicellular parasites. Functional understanding of many proteins from these pathogens has been hampered by limited sequence homology to proteins from other model organisms. Here we describe the development of a high-throughput deep mutational scanning approach in *T. brucei* that facilitates rapid and unbiased assessment of the impacts of many possible amino acid substitutions within a protein on cell fitness, as measured by relative cell growth. The approach leverages several molecular technologies: cells with conditional expression of a wild-type gene of interest and constitutive expression of a library of mutant variants, degron-controlled stabilization of I-SceI meganuclease to mediate highly efficient transfection of a mutant allele library, and a high-throughput sequencing readout for cell growth upon conditional knockdown of wild-type gene expression and exclusive expression of mutant variants. Using this method, we queried the effects of amino acid substitutions in the apparently non-catalytic RNase III-like domain of KREPB4 (B4), which is an essential component of the RNA Editing Catalytic Complexes (RECCs) that carry out mitochondrial RNA editing in *T. brucei*. We measured the impacts of thousands of B4 variants on bloodstream form cell growth and validated the most deleterious variants containing single amino acid substitutions. Crucially, there was no correlation between phenotypes and amino acid conservation, demonstrating the greater power of this method over traditional sequence homology searching to identify functional residues. The bloodstream form cell growth phenotypes were combined with structural modeling, RECC protein proximity data, and analysis of selected substitutions in procyclic form *T. brucei*. These analyses revealed that the B4 RNaseIII-like domain is essential for maintenance of RECC integrity and RECC protein abundances and is also involved in changes in RECCs that occur between bloodstream and procyclic form life cycle stages.

## 1 Introduction

*Trypanosoma brucei* species are early diverged eukaryotic, unicellular, kinetoplastid parasites that cause Human African Trypanosomiasis (also known as African Sleeping Sickness) in humans, and Nagana in domestic animals. They are closely related to *T. cruzi* and *Leishmania* parasites that cause Chagas disease and leishmaniases respectively. Studies in *T. brucei* have advanced the understanding of many fundamental biological processes and eukaryotic evolution. Indeed, a number of these processes, such as trans-splicing, polycistronic transcription, antigenic variation, glycosylphosphatidylinositol anchoring, and mitochondrial RNA editing, were first described in trypanosomes and provided novel paradigms for eukaryotic biology. However, functional studies of many proteins in *T. brucei* and related parasites have been hampered by limited sequence homology to proteins from other model organisms, which inhibits our ability to identify the amino acids most critical for function. We previously described mutagenesis and complementation screens that we used to measure the effects of different randomly generated substitutions on *T. brucei* cell fitness (McDermott et al., 2015a; Carnes et al., 2022; Davidge et al., 2023). However, these earlier screens restricted analysis to a relatively small number of substitutions due to low stable cell transfection efficiencies, in addition to complex and time-consuming cell handling procedures. Here, we report the development and application of a higher-throughput deep mutational scanning experiment in *T. brucei*. The approach enables the parallel and unbiased examination of the effects of many possible amino acid substitutions in an essential protein on cell growth and identifies amino acids with critical functions.

We applied this method to KREPB4 (B4) which is an essential protein required for mitochondrial RNA editing in *T. brucei* for which no specific function has been identified. This RNA editing process generates functional mitochondrial mRNAs via post-transcriptional insertion and deletion of uridines (Us) at numerous editing sites using guide RNA (gRNA) templates (Aphasizhev and Aphasizheva, 2011; Read et al., 2016; Cruz-Reyes et al., 2018; Aphasizheva et al., 2020). Editing reactions are catalyzed by large ~1 MDa RNA Editing Catalytic Complexes (RECCs) that contain the enzymes that perform numerous coordinated catalytic cycles of mRNA endonucleolytic cleavage, U insertion or deletion, and mRNA ligation, as well as proteins that lack known or apparent catalytic capabilities (Aphasizheva et al., 2020). Editing sites are recognized by at least three different RECCs that contain a common set of 12 proteins but differ with respect to paralogous apparently dimeric RNase III endonucleases made up of either KREN3 (N3)/KREPB6 (B6), KREN2 (N2)/KREPB7 (B7), or KREN1 (N1)/KREPB8 (B8) (Panigrahi et al., 2006; Carnes et al., 2008, 2011; McDermott et al., 2016; Carnes et al., 2022). Several other proteins that are common to the three RECC isoforms also contain single RNase III domains that lack residues essential for catalysis and are paralogs of B6-B8 and N1-N3 (Wang et al., 2003; Carnes et al., 2012; Lerch et al., 2012; McDermott et al., 2015a, b; McDermott and Stuart, 2017; Carnes et al., 2018; McDermott et al., 2019). These include B4 which we showed is essential for editing and RECC integrity (Babbarwal et al., 2007; Carnes et al., 2012; McDermott and Stuart, 2017). However, the precise roles of B4 and its RNase III-like domain in editing remain unclear. Because B4 lacks catalytic residues, understanding which amino acids are critical for its function are particularly difficult to predict, and identifying them would shed significant light on the nature of its role in editing.

Here, we used our deep mutational scanning approach to illuminate how the amino acid sequence of the B4 RNase III-like domain impacts on B4 function and bloodstream form (BF) *T. brucei* cell growth. We identified thousands of B4 variants including hundreds with single amino acid substitutions, that had various effects on cell growth and validated those with the most deleterious effects. There was no correlation between cell growth phenotypes and residue conservation, demonstrating that sequence conservation cannot reliably be used to predict function, thus highlighting the value of our deep mutational scanning method. We combined B4 structural prediction using a Discoba-specific version of AlphaFold2 (Wheeler, 2021) with modeling of RNA substrate to extend our previous homology models (McDermott et al., 2016; McDermott and Stuart, 2017). We mapped the substituted residues, their cell growth phenotypes, and our previous intra- and inter-protein amino acid proximity data (McDermott et al., 2016) onto the resulting B4-RNA structural model. This analysis revealed B4 RNase III-like domain substitutions that could potentially interfere with protein-protein interactions within RECCs, which were validated via immunoprecipitation of selected B4 variants. Finally, we show that several substitutions identified by our screen as detrimental to BF cell fitness do not affect procyclic form (PF) cell growth or RECCs. Thus, the B4 RNase III-like domain is also involved in the differences in RECCs that occur between BF and PF life cycle stages (McDermott et al., 2015a, b, 2019; Carnes et al., 2022; Davidge et al., 2023).

## 2 Materials and methods

### 2.1 Transfection and growth of *T. brucei* cells *in vitro*

BF cells were grown in HMI-9 (Hirumi and Hirumi, 1989) with 10% FBS at 37°C, 5% CO_2_. PF cells were grown in SDM-79 (Brun and Schonenberger, 1979) with 10% FBS at 27°C. Transfections of BF cell lines with the Amaxa Nucleofector (Lonza), and of PF cell lines with the BTX transfection device (Harvard Apparatus, Inc.), were carried out as described (Merritt and Stuart, 2013), with the exception that Tb-BSF buffer (90 mM sodium phosphate buffer (Na_2_HPO4/NaH_2_PO4), 5 mM KCl, 0.15 mM CaCl_2_, 50 mM HEPES pH 7.2) was used for BF nucleofection instead of the Human T Cell Nucleofector Kit (Schumann Burkard et al., 2011). Unless otherwise stated, concentrations of drugs used for selection and tetracycline (tet)-regulated expression of transgenes are as follows. For BF: 2.5 µg/mL G418, 5 µg/mL hygromycin, 2.5 µg/mL phleomycin, 0.5 µg/mL tet, 0.1 µg/mL puromycin, 5 µg/mL blasticidin, 12 µg/mL nourseothricin, 29 µg/mL trimethoprim. For PF: 15 µg/mL G418, 25 µg/mL hygromycin, 2.5 µg/mL phleomycin, 0.5 µg/mL tet, 1 µg/mL puromycin, 10 µg/mL blasticidin.

### 2.2 Mutant B4 *T. brucei* library development and generation

#### 2.2.1 Error-prone PCR for mutant domain library generation

Error-prone PCR mediated mutagenesis of 249 bp spanning the B4 RNase III-like domain was performed using the GeneMorph II EZClone Domain Mutagenesis kit (Agilent Technologies). The template plasmid was the Gateway entry clone pDONR221-B4 which contains the wild-type (WT) B4 open reading frame (ORF) without the stop codon. Briefly, 100 ng of the target domain DNA (15.2 μg template plasmid) was mutagenized by 30 cycles of PCR according to the manufacturer’s protocol using the following primers: 5’-TTCCTGGGCGAAAGCTTT-3’ and 5’-GAGAACATTTGCAACTCCCC-3’ (**Supplementary Table 1**). The PCR products were separated by gel electrophoresis and purified using a gel-extraction kit (Qiagen). The purified mutagenized PCR products were then used as megaprimers for amplification (25 cycles) of pENTR-Express-B4 plasmid which contains the WT B4 ORF without the stop codon flanked by attL sites, and in frame with the neomycin phosphotransferase sequence that confers resistance to kanamycin (Gray et al., 2007).

#### 2.2.2 Full-length B4 allele selection and pENTR-Express allele library isolation

2 x 100 μL of ElectroMAX DH10B Electrocompetent cells (ThermoFisher Scientific) were each transformed with 500 ng of the pENTR-Express-B4-mutated domain library using a BTX ECM 630 electroporator (settings: 1700 V, 200 MΩ, 25 μF). Cells from each transformation were recovered for 1 h in 1 mL SOB + 1 mM IPTG at 37°C at 250 rpm. An aliquot of each transformation was serially diluted, plated on LB plates containing 30 μg/mL kanamycin and 1 mM IPTG, and incubated 30°C for 36 h to titer the number of Kan^+^ colonies, while the remainder of each transformation was stored as a glycerol stock. The optimal kanamycin concentration of 30 μg/mL for selection of full-length B4 in the pDONR-Express system was determined as previously described (McDermott and Stuart, 2017). Glycerol stocks were thawed on ice and plated out on 245 mm LB plates containing 30 μg/mL kanamycin and 1 mM IPTG at a density of ~20,000 colonies/plate to produce an overall number of ~200,000 Kan^+^ colonies. Plates were incubated at 30°C for 36 h, all colonies were scraped from plates, and plasmid DNA was isolated using the QIAfilter midiprep kit (Qiagen).

#### 2.2.3 Transfer of the mutant allele library into a plasmid for constitutive expression in *T. brucei*

500 ng of the destination vector pHD1344tub(PAC)GW-Cterm3V5 (McDermott et al., 2015b), 250 ng of pENTR-Express containing the mutated domain B4 allele library, 2 μL of LR Clonase II enzyme mixture (Life Technologies), and TE to 10 μL were incubated at room temperature (25°C) for 20 h. The reaction was stopped by adding 1 μL Proteinase K and incubating at 37°C for 10 min. 2 μL of the LR reaction was transformed into each of 5 x 100 μL of ElectroMAX DH10B Electrocompetent cells (ThermoFisher Scientific) as described above. Cells were recovered for 1 h in 1 mL of SOB at 37°C at 250 rpm. Serial dilutions were performed and plated on LB plates containing 100 μg/mL ampicillin to titer, with the remainder of the transformation stored as a glycerol stock. Plates were incubated at 37°C for 20–24 h. After the titer was determined, the glycerol stock was thawed on ice and plated on 245 mm LB plates containing 100 μg/mL ampicillin at a density of 30,000 colonies/plate to produce an overall number of 150,000 Amp^+^ colonies. Plates were incubated at 37°C for 24 h, all colonies were scraped from plates, and plasmid DNA was isolated using the QIAfilter midiprep kit (Qiagen). The resulting expression vector library was designated pHD1344tub(PAC)-mutRIIIKREPB4-Cterm3V5. This plasmid library contains the puromycin resistance selectable marker and allows for constitutive expression of C-terminally 3xV5 tagged mutant library alleles in the β-tubulin locus.

#### 2.2.4 Development of a trimethoprim-inducible I-SceI meganuclease system to increase BF *T. brucei* transfection efficiency

A β-tubulin-targeted construct containing 1) ddDHFR-tagged I-SceI flanked by GPEET 5’ and ribosomal protein L4 3’UTRs, 2) an embedded I-SceI cleavage site, and 3) nourseothricin resistance (NAT) and herpes simplex virus-thymidine kinase (HSVTK) flanked by aldolase 5’ and 3’UTRs, was sequentially generated between the HindIII and Bsu36I sites in pHD1344tub(PAC) (Carnes et al., 2012). The ddDHFR-tagged I-SceI sequence was obtained by PCR amplification from pEVL3 (a gift from Phillip Yates) and cloned into the HindIII and BamHI sites of pHD1344tub(PAC). The NAT-HSVTK sequence was generated first by cloning the NAT open reading frame from pYL16 (Werner BioAgents) between the BglII and XbaI sites of pyrFEKO-BSD (pSM06) (McDermott et al., 2015b). NAT-HSVTK was then amplified from the resulting pyrFEKO-NAT plasmid and cloned between the SpeI and Bsu36I sites within pHD1344tub(PAC)-SceI-ddDHFR. A ribosomal protein L4 3’UTR, I-SceI cleavage site, and aldolase 5’UTR cassette was generated by overlap extension PCR and cloned between the BamHI and SpeI sites of pHD1344tub(NAT-HSVTK)-SceI-ddDHFR. All primers are described in **Supplementary Table 1**. 10 μg of the resulting pHD1344tub(NAT-HSVTK)-Sce1CS-SceI-ddDHFR plasmid was linearized with *Not*I and transfected into 3 x 10^7^ BF B4 CN cells using the Amaxa Nucleofector (McDermott and Stuart, 2017). Transgenic lines were selected by nourseothricin resistance and correct insertion of the I-SceI construct assessed by PCR. Three independent cell lines were then transfected in triplicate with pHD1344tub(PAC)-KREPB4 in the presence of 0, 10, and 100 µM trimethoprim added 6 h prior to transfection. Cells where the B4 allele had replaced the I-SceI-ddDHFR cassette were selected by puromycin and ganciclovir resistance for estimation of transfection efficiency.

#### 2.2.5 RNA isolation and RT-qPCR analysis

Total RNA was harvested using TRIzol and treated with TURBO DNase (Life Technologies) according to manufacturer’s instructions. RNA integrity was confirmed using an RNA nanochip on a BioAnalyzer (Agilent Technologies). 2 µg of total RNA was reverse transcribed using TaqMan Reverse Transcription Reagents and MultiScribe Reverse Transcriptase (Life Technologies). The abundance of TERT reference and B4 transcript cDNAs were then analyzed by realtime PCR (Carnes and Stuart, 2007) using the QuantStudio 3 system (ThermoFisher Scientific). Primers are described in **Supplementary Table 1**. Calculations of RNA levels in samples following tet withdrawal (for 48 hours in BF), relative to the presence of tet, were done using the 2 [-ΔΔC(T)] method (Livak and Schmittgen, 2001) using TERT as an internal reference. Technical duplicates of each cDNA sample were assayed for each target and internal reference per experiment and C(T) data averaged before performing the 2 [-ΔΔC(T)] calculation. Experiments were repeated using three biological replicate independent cell lines.

### 2.3 Protocols for conducting and validating screen

#### 2.3.1 Functional selection of library alleles

The minimum concentration of tet required for parental BF B4 CN cell survival was determined by growth in a range of tet concentrations (0, 1, 2, 5, 10, 50, 500 ng/mL) (**Supplementary Figure 1**). 5 ng/mL was determined to be the minimum required for regulated WT B4 expression and was used throughout to ensure full tet withdrawal by dilution during screening. Briefly, 5 x 10^7^ BF B4 CN cells containing pHD1344tub(NAT-HSVTK)-Sce1CS-SceI-ddDHFR were treated with 100 µM trimethoprim for 6 h to induce I-SceI-mediated cleavage of the target locus, then transfected with 12.5 μg of *Not*I-linearized pHD1344tub(PAC)-mutRIIIKREPB4-Cterm3V5 plasmid containing the mutant library using the Amaxa nucleofector. Following transfection, cells were diluted into 300 mL HMI-9 medium containing 5 ng/mL tet. Cells were allowed to recover for 6 h before addition of puromycin and ganciclovir for selection. An aliquot of cells was also taken following transfection and serially diluted in media containing puromycin and ganciclovir to estimate transfection efficiency and library complexity. Two separate transfections of the mutant library into independent I-SceI expressing cell lines were carried out with an efficiency of ~2.3 x 10^-3^, yielding approximately 100,000 and 120,000 transfectants per library. Following 6 days of selection and growth to a total of >1 x 10^8^ cells, genomic DNA (gDNA) was harvested from 5 x 10^7^ cells per library transfection (>400x of each transfectant) as our input samples. Another 5 x 10^7^ cells were then washed to remove tet and resuspended in 175 mL HMI-9 and grown for 4 days as described above. During growth, the total cell numbers in our BF library pools were always kept above 5 x 10^7^, with density below 2 x 10^6^ cells/mL to maintain library complexity and growth in logarithmic phase. gDNA was harvested from 5 x 10^7^ cells on day 4 following tet withdrawal, reflecting the time-point at which expression of tet-regulated WT B4 is robustly repressed and growth defects are observed in the parental BF B4 CN cells (**Supplementary Figures 2A, B**) (McDermott and Stuart, 2017).

#### 2.3.2 PCR amplification and sequencing of library alleles

gDNA extraction was carried out using the NucleoSpin Blood kit (Macherey-Nagal), eluting in a volume of 100 µL. A total of 24 cycles of PCR amplification using Q5 High-Fidelity DNA Polymerase (NEB) were used to amplify the mutated domain and add sequencing primer binding sites, indices, and P5/P7 flow cell attachment sites for Illumina sequencing. To ascertain the range of linear amplification for specific primer sets (**Supplementary Table 1**), real-time PCR using SsoAdvanced Universal SYBR Green Supermix (BioRad) was performed (not shown). The final amplicon size was 423 bp. PCR products were visualized and purified by electrophoresis on FlashGel DNA cassettes (Lonza).

PCR amplicon concentrations were determined using a Qubit Fluorometer (ThermoFisher Scientific), and molar concentrations calculated using the final amplicon size. Equimolar amounts of all indexed amplicons were then combined into a single library for analysis on the Illumina MiSeq. All amplicons were sequenced together on a single flow cell to eliminate the possibility of uncontrolled variability between sequencing runs.

#### 2.3.3 Data analysis

Fitness estimates were computed with DiMSum v0.3.2.9000 (https://github.com/lehner-lab/DiMSum) (Faure et al., 2020), which derives final fitness estimates as an error-weighted sum of replicate fitness values, after computing wildtype-normalized fold changes at the replicate level. DiMSum was run with the following TRIM, ALIGN, and PROCESS arguments: cutadaptErrorRate=0.4 vsearchMinQual=28, indels=none, maxSubstitutions=9, mixedSubstitutions=T, and fitnessMinInputCountAll=50. Data were visualized and plotted onto a predicted and modeled B4 structure (see below) using dms-view (Hilton et al., 2020).

#### 2.3.4 Screen validation via generation of independent exclusive expression cell lines

The Gateway expression clone pHD1344tub(PAC)-KREPB4-Cterm3V5 (McDermott and Stuart, 2017) was used as a template for site-directed mutagenesis (QuikChange II kit; Agilent) using the primers listed in **Supplementary Table 1**. NotI-digested plasmids were transfected into the relevant BF and PF B4 CN cells (McDermott and Stuart, 2017). Transfections of BF cell lines with the Amaxa Nucleofector (Lonza) and of PF cell lines with the BTX transfection device (Harvard Apparatus, Inc.) were carried out as described above. Cell lines resistant to puromycin were selected, and constitutive expression of B4-3×V5 was confirmed by Western blotting. For standard growth curve analyses of independent cell lines, cell density + and - tet were measured using a Coulter Counter. BF were reseeded at 0.75 x 10^5^ cells/mL in 10 mL every day, whilst PF were reseeded at 1 x 10^6^ cells/mL in 10 mL every two days. To generate cell growth heat maps, cumulative growth numbers for - tet and + tet cultures were calculated for each time point. Next, the log2 of the ratio of - tet to +tet was calculated, and this number was converted to blue to orange scale using conditional formatting in Microsoft Excel.

### 2.4 Structural predictions and protein structure modelling

The structure of B4 was predicted using a ColabFold notebook-based version of AlphaFold2/MMseqs2 that incorporates modifications for analyzing proteins from organisms within the Discoba clade that include Trypanosoma (Steinegger and Soding, 2017; Wheeler, 2021). Unstructured and disordered regions of very low (AlphaFold pLDDT score < 50) and low (AlphaFold pLDDT score < 70) confidence at the N- and C termini of B4 were removed for clarity.

Predicted structures were modeled onto *Saccharomyces cerevisiae* Rnt1p RNase III crystal structure (5T16) using the Matchmaker function in Chimera (Pettersen et al., 2004). Modeling parameters are described in **Supplementary Data**. Prediction of contacts between B4 and modeled dsRNA, as well as crosslink visualization and distance measurements on predicted structures and models were also performed using Chimera.

### 2.5 Immunoprecipitation, SDS-PAGE, and western blotting

Cleared lysate was prepared by lysis of 6 × 10^8^ BF or 4 × 10^8^ PF cells in 1.0 mL IPP150 (10 mM Tris-HCl, pH 8.0, 150 mM NaCl, 1% Nonidet P-40) with 1% Triton X-100, followed by centrifugation at 10,000 × g and 4°C. For each immunoprecipitation, 0.5 mL cleared lysate (3 × 10^8^ BF or 2 × 10^8^ PF cells) was incubated overnight with 2 μL of rabbit antibody specific for the V5 epitope tag (Rockland Immunochemicals; #600-401-378). Magnetic beads (25 μL; Protein G Mag Sepharose Xtra; GE Healthcare) were washed twice with 1 mL of 1× phosphate-buffered saline–0.1% bovine serum albumin and once with 1 mL IPP150. The beads were then incubated for 4 h with rotation at 4°C with cleared lysate/antibody. After incubation, the supernatant was removed, and the beads were washed four times with 1 mL of IPP150. Complexes bound to beads were eluted by heating with 100 μL of 2× SDS sample buffer for 5 min at 95°C. Samples containing purified protein complexes were resolved on 10% SDS-polyacrylamide gels (Criterion Tris-HCl; Bio-Rad). For Western blotting, resolved proteins were transferred to Immobilon-P polyvinylidene difluoride membranes (Millipore) and probed using mouse monoclonal antibodies against KREPA1, KREPA2, KREL1, and KREPA3 as previously described (Panigrahi et al., 2001). Blots were sequentially stripped and reprobed using mouse monoclonal primary antibody against the V5 epitope tag (Thermo Fisher Scientific; #R960-25) at 1:5,000 with goat anti-mouse immunoglobulin secondary antibody conjugated with horseradish peroxidase at 1:5,000. Blots were developed with an enhanced chemiluminescence kit (Thermo Scientific) per the manufacturer’s instructions and imaged using X-ray film (Kodak).

## 3 Results

### 3.1 Generation and high-throughput screening of a mutated domain allele library in *T. brucei*

**Figure 1:**
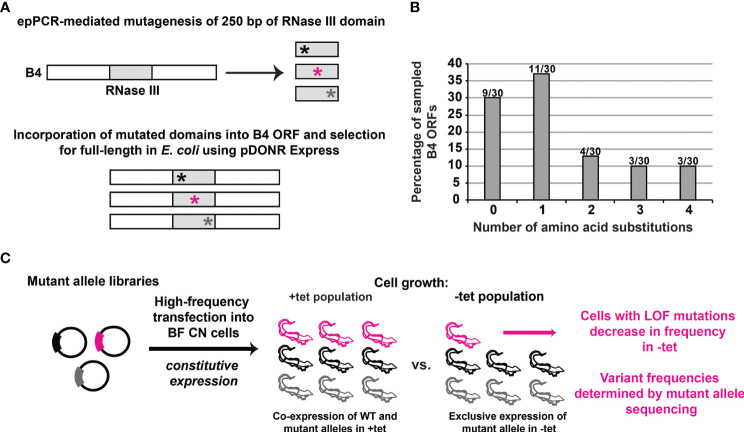
High-throughput identification of loss-of-function mutations in a protein domain of interest in BF *T. brucei*. **(A)** Diagram showing that the KREPB4 (B4) RNase III-like domain was mutagenized by error-prone (ep)PCR (represented by asterisks) and placed in frame within the B4 ORF. Nonsense and frameshift mutations were eliminated by selection in *E. coli* using pDONR Express before transfer to a *T. brucei* vector for constitutive expression from the tubulin locus (Gray et al., 2007; McDermott et al., 2015a; Carnes et al., 2022; Davidge et al., 2023). **(B)** 30 alleles were analyzed following library generation to assess the distribution of number of amino acid substitutions per *in silico* translated ORF. Columns indicate percentage of the 30 sampled alleles containing given numbers of amino acid substitutions. Numbers above the columns are the number of alleles per number of amino acid substitutions. **(C)** Schematic of the genetic complementation assay used to identify the loss-of-function (LOF) substitutions in BF *T. brucei*. The mutant plasmid library generated as shown in panels A and B was transfected at high frequency for constitutive expression in BF B4 CN cells. The population of transformed cells was grown in the presence of tet for expression of regulatable WT B4, and in the absence of tet for repression of regulatable WT B4 and exclusive expression of the mutant variants. Cells containing LOF variants do not grow and therefore decrease in frequency in the population in the absence of tet. High-throughput DNA sequencing is used to measure the frequency of each variant in the absence vs. presence of tet.

We sought to establish a high-throughput complementation assay for scoring the function of multiple protein variants in *T. brucei.* We focused on the RNase III-like domain of B4 and used our BF B4 CN cell lines, in which both endogenous B4 alleles have been deleted, and in which a tet-inducible WT B4 allele has been inserted into the rRNA locus (McDermott and Stuart, 2017) (**Figures 1A–C**). First, we created a library of alleles in which 249 bases encoding 83 amino acids spanning the B4 RNase III-like domain were randomly mutated using error-prone PCR (**Figure 1A**). The library was enriched for full-length alleles in *E. coli* as previously described (Gray et al., 2007; McDermott et al., 2015a), and subcloned into a vector for constitutive expression from the tubulin array in *T. brucei*. Approximately 150,000 plasmids were present in the final *T. brucei* expression construct library. Thirty individual alleles were sequenced which revealed an average of 2.3 amino acid substitutions per encoded variant. Importantly, the plurality (37%) contained just a single amino acid substitution, 50% contained either one or two substitutions, and 30% encoded wild-type (WT) variants that did not contain any substitutions (i.e., had no or only silent mutations) (**Figure 1B**). All sampled alleles also encoded full-length variants.

**Figure 2:**
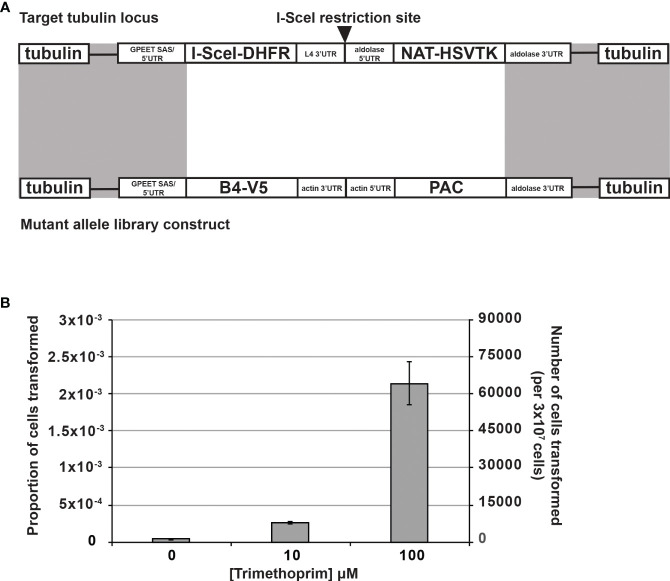
Stabilization of I-SceI with the dihydrofolate reductase destabilization domain (ddDHFR) by trimethoprim increases BF transfection efficiency. **(A)** An I-SceI-ddDHFR expression construct with an embedded I-SceI site was transfected for constitutive expression into the tubulin locus of BF B4 CN cells, providing a genomic target site for high frequency homologous recombination of mutant library constructs (recombination sites shaded gray) (Glover and Horn, 2009). The I-SceI-ddDHFR protein is stabilized only upon addition of trimethoprim leading to I-SceI site cleavage and double strand break (DSB) induction, which in turn stimulates repair via homologous recombination using the mutant construct. NAT and PAC genes allow for positive cell selection in nourseothricin and puromycin respectively. Herpes simplex virus-thymidine kinase (HSVTK) allows for negative selection in ganciclovir. **(B)** I-SceI and DSB induction in the cells described in **(A)** by trimethoprim increases transfection efficiency of mutant library constructs in a dose-dependent manner. Experiment was carried out in triplicate with three independent clonal cell lines containing the I-SceI-ddDHFR expression construct.

To increase the efficiency and reproducibility of transfection of our large allele library into our BF CN cells, we developed an inducible I-SceI meganuclease-based system to introduce double-strand breaks at a single β-tubulin locus (Alsford et al., 2005; Glover et al., 2007, 2008; Glover and Horn, 2009; Alsford et al., 2011; Glover et al., 2015). Because our CN cell line already contained a tet-regulated WT B4 construct, we used a trimethoprim (TMP)-stabilized dihydrofolate reductase (DHFR) destabilizing domain (ddDHFR) to regulate I-SceI expression (Iwamoto et al., 2010; Rakhit et al., 2011; Ma et al., 2015; Podesvova et al., 2017). Briefly, we integrated a construct containing ddDHFR-tagged I-SceI, an embedded I-SceI cleavage site, and nourseothricin and herpes simplex virus-thymidine kinase (HSVTK) selectable markers into a β-tubulin locus of BF B4 CN cells (**Figure 2A**). The resulting I-SceI construct containing cell lines respond to tet-withdrawal with the same kinetics as the parental BF B4 CN cells (McDermott and Stuart, 2017) i.e., levels of B4 mRNA are reduced by >97% 2 days following tet withdrawal, resulting in growth inhibition that is apparent 3 days following tet withdrawal (**Supplementary Figures 2A, B**). To test the system, three independent BF B4 CN cell lines containing the I-SceI construct were treated in triplicate with increasing concentrations of TMP at 0, 10, and 100 µM for 6 h prior to transfection of a second β-tubulin-targeted construct containing a WT B4 allele and puromycin selectable marker. Following transfection, cells were positively selected in puromycin and negatively selected in ganciclovir. The approach increased transfection efficiency in a TMP dose-dependent manner, and by approximately two orders of magnitude from ~4.2 x 10^-5^ to ~2.1 x 10^-3^ upon treatment with 100 µM TMP (**Figure 2B**), generating up to ~70,000 unique cell lines in transfections of 3 x 10^7^ cells (**Table 1**). Sequence analysis and growth of the resulting cell lines both in the presence and absence of tet (**Supplementary Figure 2C**) showed that site-specific cleavage at the tubulin locus had promoted the desired integration of the WT B4 allele, where it had replaced the I-SceI construct.

**Table 1:** Stabilization of I-SceI with the DHFR destabilization domain (ddDHFR) by trimethoprim increases BF transfection efficiency.

Cell line	[Trimethoprim] μM	Transfection efficiency (proportion of cells transformed)	Number of transformed cells per transfection of 3x10^7^ cells
CN	0	3.1 x 10^-5^	930
CN	100	3.0 x 10^-5^	900
CN + SceI a R1	0	4.1 x 10^-5^	1230
CN + SceI a R2	10	2.6 x 10^-4^	8100
CN + SceI a R3	100	2.3 x 10^-3^	69995
CN + SceI b R1	0	4.1 x 10^-5^	1230
CN + SceI b R2	10	2.8 x 10^-4^	8400
CN + SceI b R3	100	2.3 x 10^-3^	69625
CN + SceI c R1	0	4.6 x 10^-5^	1390
CN + SceI c R2	10	2.4 x 10^-4^	7400
CN + SceI c R3	100	1.8 x 10^-3^	54000

CN = conditional null cells; CN + SceI a, b, or c = independent CN cell lines a, b, and c, containing the I-SceI-ddDHFR expression construct with an embedded I-SceI site; R1, 2, or 3 = experimental replicate.

**Figure 3:**
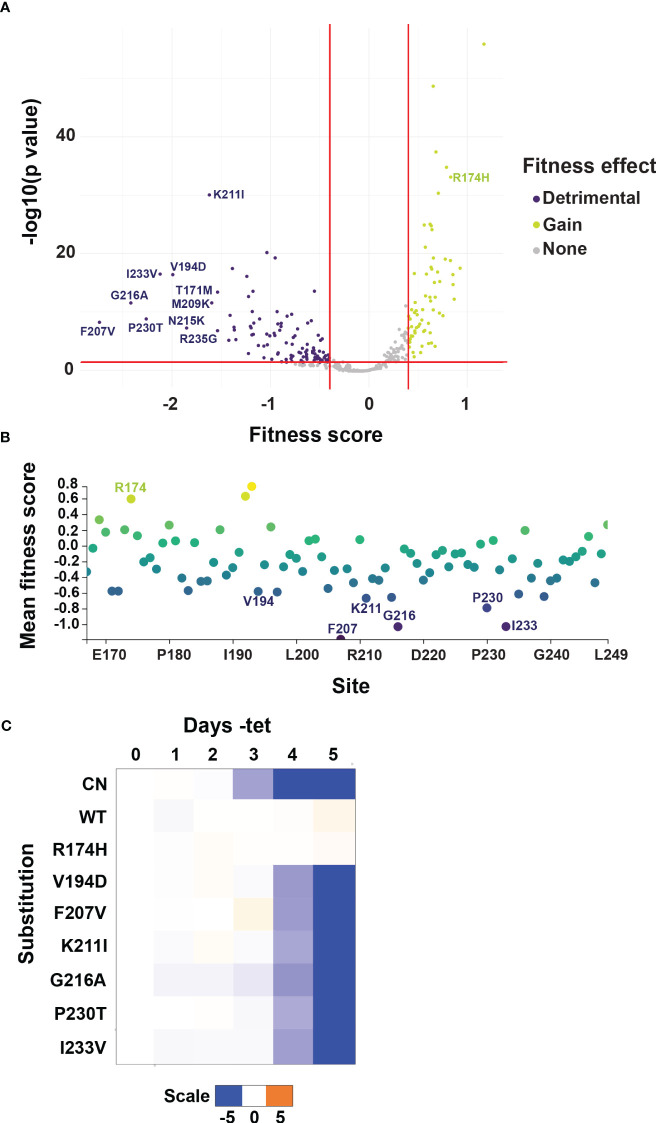
Identification of LOF substitutions in B4 by mutational scanning. **(A)** Volcano plot showing substitution-level fitness scores for variants containing single amino acid substitutions. Fitness scores are based on changes in frequency from input cell populations grown in the presence of tet to selected cell populations grown in the absence of tet and serve as a proxy for the function of each variant. Red lines and colored variants indicate fitness scores above or below 0.4 or -0.4 respectively, corresponding to wildtype-normalized fold changes of 1.5, and p values < 0.05. The ten substitutions with the most negative fitness scores i.e., most detrimental to BF growth, are labeled. **(B)** Mean site-level fitness scores for all single amino acid substitutions at each mutated site. Color on plot reflects score value, with positive scores yellow/green, and negative scores blue/purple. Sites where individual variants had the most detrimental fitness effects in **(A)** are labeled. **(C)** Alleles encoding selected single amino acid LOF or control R174H substitutions identified by mutational scanning were recreated by site-directed mutagenesis and transfected into the BF B4 CN cell line for growth phenotype validation. The log2 ratio of the effect on cumulative growth in absence versus presence of tet is indicated by the scale showing reduction in blue, increase in orange, and no effect on growth in white.

The mutated RNase III-like domain allele library was transfected into two independent I-SceI expressing BF B4 CN cell lines. Serial dilution revealed that we obtained libraries containing approximately 100,000 and 120,000 transfected cells respectively. Each of the *T. brucei* libraries were grown to logarithmic phase in the presence of tet and then diluted into media that lacked tet. Since B4 mRNA levels are significantly reduced by 2 days, and cell growth is inhibited by 3 days, following tet withdrawal (**Supplementary Figures 2A, B**) we collected cells both before (input; plus tet) and after 4 days of growth minus tet. We extracted genomic DNA, PCR amplified the segment that had been mutated, and carried out Illumina sequence analysis for each time-point. We identified 75,879 distinct nucleotide sequences, including the WT nucleotide sequence and sequences containing between 1-10 nucleotide changes, across all time-point and replicate samples. Of the 75,878 sequences that had nucleotide changes, 3,303 contained silent mutations which did not encode any amino acid substitutions, 20,909 encoded sequences with a single amino acid substitution (735 unique amino acid sequences), 34,907 had two amino acid substitutions (27,619 unique amino acid sequences), and the remaining 16,759 had between three and nine amino acid substitutions from WT sequence (15,708 unique amino acid sequences) (**Supplementary Table 2**). After filtering variants based on having a minimum read count of 50 in both replicate input libraries generated from cells grown in the presence of tet (**Supplementary Figure 3**), we calculated fitness scores based on changes in frequency from input plus tet to minus tet cell populations (Faure et al., 2020). These fitness scores serve as a proxy for cell growth and thus function of each variant. Variant fitness scores were highly correlated in our replicate experiments (**Supplementary Figure 4**). The filtered dataset quantifies the effects of 350 single amino acid changes on cell growth with high reproducibility, which we focused on to allow for easier deconvolution of mutant phenotypes (**Figure 3** and **Supplementary Table 3**). Within this group we identified substitutions at every site in the 83 amino acid mutated region, with on average ~4 different substitutions at each position. Of these, 102 single substitutions at 61 of the 83 sites had a detrimental effect i.e., fitness score below -0.4 and p value < 0.05, a further 183 single substitutions in 77 of the 83 amino acids had no significant effect, and 65 single substitutions in 44 amino acids appeared to result in a slight beneficial effect on cell fitness (**Figure 3A**). We calculated a mean site-level fitness score using the fitness scores for all individual substitutions per amino acid position (**Figure 3B** and **Supplementary Table 3**). Perhaps unsurprisingly, residues with the lowest i.e., most detrimental, mean fitness scores were also sites of the lowest individual substitution fitness scores (**Figures 3A, B**). We confirmed that selected single substitutions were responsible for the growth defects observed in our high-throughput complementation screens by recreating WT and site-directed V5-tagged mutant constructs and transfecting them into the parental BF CN cell line (**Figure 3C** and **Supplementary Figure 5**). Expression of the WT or mutant alleles was confirmed by Western analysis that probed for the V5 tag (**Supplementary Figure 5**). Growth analyses confirmed the fitness phenotypes of selected detrimental loss-of-function (LOF) substitutions, as well as of control substitutions that did not result in growth defects in our screens (**Figure 3C**, **Supplementary Figure 5**, and **Supplementary Data**).

### 3.2 Comparison of variant fitness scores to evolutionary conservation

**Figure 4:**
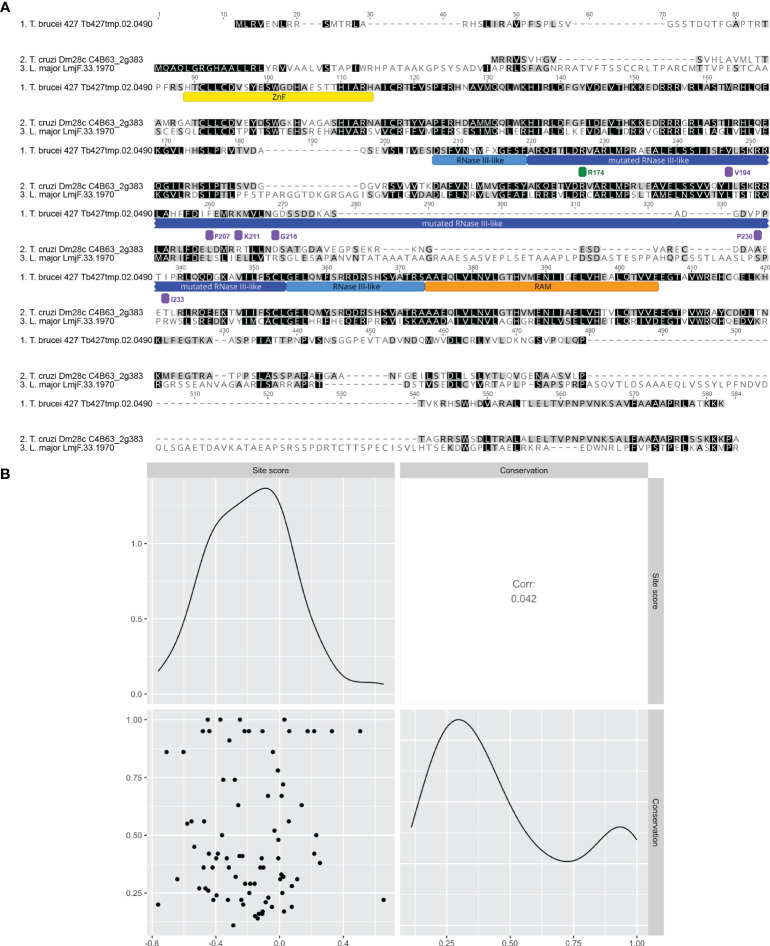
Site-level fitness score is not correlated with the level of conservation in B4. **(A)** Clustal Omega alignment of *T. brucei* B4 with representative orthologs from *T. cruzi* and *L. major*. Mutated region of RNase III-like domain underlined with dark blue. Other regions are underlined as follows: full RNase III-like domain boundaries with light blue, zinc finger (ZnF) with yellow, and RNase III Associated Motif (RAM) with orange. Positions of mutated or control sites identified as having validated detrimental or no fitness effects are indicated by bars colored to indicate mean fitness score as in **Figure 3**. **(B)** Scatterplot matrix depicting correlations between mean site-level fitness and conservation scores per site in the mutated region (labelled site score and conservation respectively). Conservation score was calculated for each amino acid as mean pairwise identity over all pairs per column in the full alignment of *T. brucei* B4 with 41 orthologs from a range of kinetoplastid species including *T. cruzi* and *L. major* (see **Supplementary Figure 6** for full alignment). Upper-right and lower-left matrix cells show Pearson correlation coefficient and scatterplot respectively. Lower-right and upper-left matrix cells are density plots showing the distribution of values used in the analysis for conservation score and mean site-level fitness score respectively.

Conservation analysis is a widely used method for prediction of sites that are important for protein function, as mutational sensitivity and evolutionary conservation are often strongly correlated (Stone and Sidow, 2005). To study the correlation between B4 RNase III-like domain conservation and function as defined by our screen, we quantified the degree of conservation for each B4 amino acid position across a wide range of orthologs from 42 kinetoplastid species including *T. cruzi* and *L. major* (**Figure 4A** and **Supplementary Figure 6**). We then calculated a conservation score for each site as the mean pairwise identity over all pairs per column in an alignment of all orthologs (**Supplementary Table 3**). Calculation of the Pearson correlation coefficient between conservation and site-level fitness scores revealed no appreciable linear correlation (**Figure 4B**), demonstrating that B4 sequence conservation cannot be solely used to predict function. Considerable variation in correlation between conservation and variant effects has been observed across previous deep mutational scanning experiments in other model systems and is protein dependent (Hoie et al., 2022), further highlighting the power of this mutational scanning approach.

### 3.3 Mapping detrimental substitutions onto a predicted B4 structure

**Figure 5:**
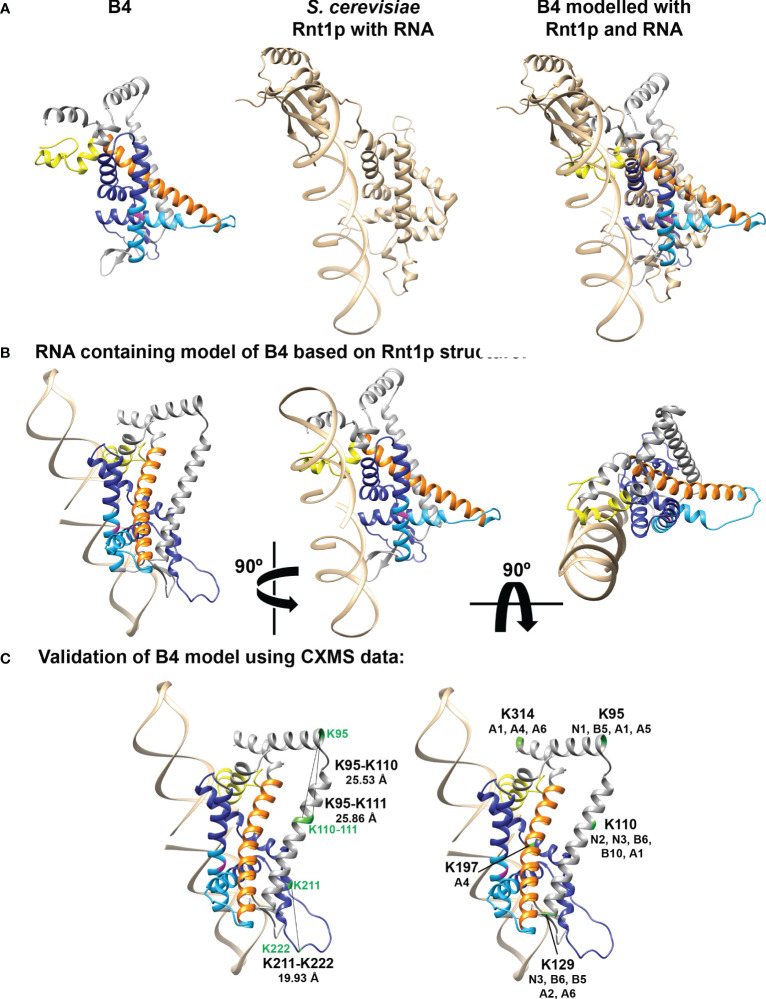
Model of predicted B4 structure and comparison with known RNase III structures. **(A)** B4 structure was predicted using AlphaFold2 with modifications for analyzing proteins from organisms within the Discoba clade (Wheeler, 2021) and shown alongside the crystal structure of *S. cerevisiae* Rnt1p RNase III monomer with bound RNA (PDB structure 5T16; shown in tan). Mutated region of RNase III-like domain is shown in dark blue, full RNase III-like domain boundaries shown in light blue, ZnF shown in yellow, and RAM shown in orange. Universally conserved RNase III signature motif glycine is highlighted in magenta. The B4 and Rnt1p structures were overlaid using the Matchmaker function in Chimera, colored as described above. **(B)** Three views rotated by 90° of the model of B4 monomer with RNA based on the predicted B4 Discoba AlphaFold2 structure and the crystal structure of the *S. cerevisiae* Rnt1p RNase III dimer with RNA substrate (PDB structure 5T16). Model colors as in **(A)**. **(C)** Mapping of intra- and inter- crosslink positions within B4 and between B4 and other RECC proteins, onto the predicted B4 structure. Model colors as in **(A)**. Crosslinked residues from (McDermott et al., 2016) are shown in green.

The release of AlphaFold2 and Discoba-specific improvements (Wheeler, 2021) together provide powerful resources to predict *T. brucei* B4 structure and to understand the effects of our amino acid substitutions on B4 RNase III function (**Supplementary Figure 8**). For clarity, we removed low confidence regions at the N and C-termini of the modelled B4 that were unstructured i.e., pLDDT score < 70 (**Figure 5A**, **Supplementary Table 3**, and **Supplementary Figure 7**). These low confidence regions also correlate with regions of predicted disorder, particularly in the mutated RNase-like domain (**Supplementary Table 3** and **Supplementary Figure 8**) (Horvath et al., 2020; Miskei et al., 2020; Hatos et al., 2022, 2023). Comparison with the crystal structures of archetypal eukaryotic (*Saccharomyces cerevisiae* Rnt1p; PDB 5T16) (**Figure 5A**) and bacterial (*Aquifex aeolicus* RNase III; PDB 2NUF) (not shown) RNase III proteins confirmed the presence of the RNase III domain-like fold in B4, as expected from our previous sequence searches and homology modelling (22,46,47). Furthermore, comparisons with the AlphaFold predicted structures of other RECC RNase III paralogs that all contain an RNase III Associated Motif (RAM) flanking their RNase III or RNase III-like domains (Carnes et al., 2022), revealed that B4 also has a RAM. We modelled the Discoba AlphaFold2 predicted structure for B4 onto the *S. cerevisiae* Rnt1p RNase III crystal structure and built a dsRNA substrate-containing model (**Figures 5A, B**). We further validated our model using our previous BS3 crosslinking mass spectrometry data (22). BS3 has a linker arm of 11.4 Å when fully extended and can crosslink two residues whose Cα atoms are up to 30 Å apart (48). We measured the distances between the B4 residues that could be mapped onto the predicted structure and model. We were able to measure the distances for three intralinks (**Figure 5C** and **Supplementary Table 4**). The distances between crosslinked residues were <30 Å, indicating that the model is consistent with available experimental data and provides a reasonable representation of the structure. We also highlighted B4 residues that crosslinked with other RECC proteins on our model (**Figure 5C** and **Supplementary Table 5**). These analyses did not identify crosslinks to other proteins in the vicinity of the modelled dsRNA but did identify several crosslinks to other RECC RNase III and OB-fold proteins in other regions.

**Figure 6:**
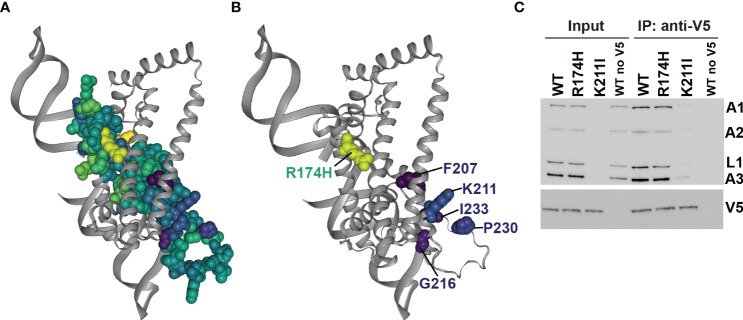
Mapping of substitutions onto the B4 structure model. **(A)** All amino acids that were substituted in the mutational scanning screen are shown as space-filled residues on the predicted B4 Discoba AlphaFold2 structure and colored according to mean fitness score as in **Figure 3B**. **(B)** As **(A)**, but only residues with the five most detrimental mean fitness scores in the mutational scanning screen are shown as space-filled residues. **(C)** BF cells that exclusively expressed V5-tagged WT or mutant B4, or untagged WT B4 control following two days of repression of the regulatable WT B4 allele were used for anti-V5 tag immunoprecipitation of B4-bound protein complexes. Cleared input cell lysates (5%) and anti-V5 immunoprecipitates (10%) were analyzed by Western blotting. Blots were probed with monoclonal antibodies against RECC proteins KREPA1, KREPA2, KREL1 and KREPA3, and anti-V5 antibody.

To assess fitness and protein function in the context of structure, we mapped the mean site-level fitness scores onto our structural model (**Figures 6**, **3B**, and **Supplementary Table 3**) (Hilton et al., 2020). Comparison with the previous protein-protein crosslinking data (**Supplementary Figure 9**) (McDermott et al., 2016) revealed detrimentally substituted residues that are proximal to regions implicated in RECC protein binding, and therefore that potentially interfere with B4 protein-protein interactions within RECCs. We tested this via immunoprecipitation of V5-tagged detrimental K211I and control R174H mutant B4 (**Figure 6B** and **Supplementary Figure 5**). K211 is close to sites of B4 interlinks with other RECC proteins (K129 and K110) and intralinks within B4 (**Supplementary Figure 9**). As observed previously for cells that lack B4 (McDermott and Stuart, 2017), cells that exclusively expressed the K211I B4 variant had much-reduced signals for other RECC components in input and co-immunoprecipitation compared to cells exclusively expressing WT B4 or the control R174H variant (**Figure 6C**). We interpret the reductions in total levels of RECC components as due to turnover of proteins that cannot be incorporated into RECCs without functional B4.

### 3.4 Life-cycle stage differences in fitness of cells containing substitutions in B4

**Figure 7:**
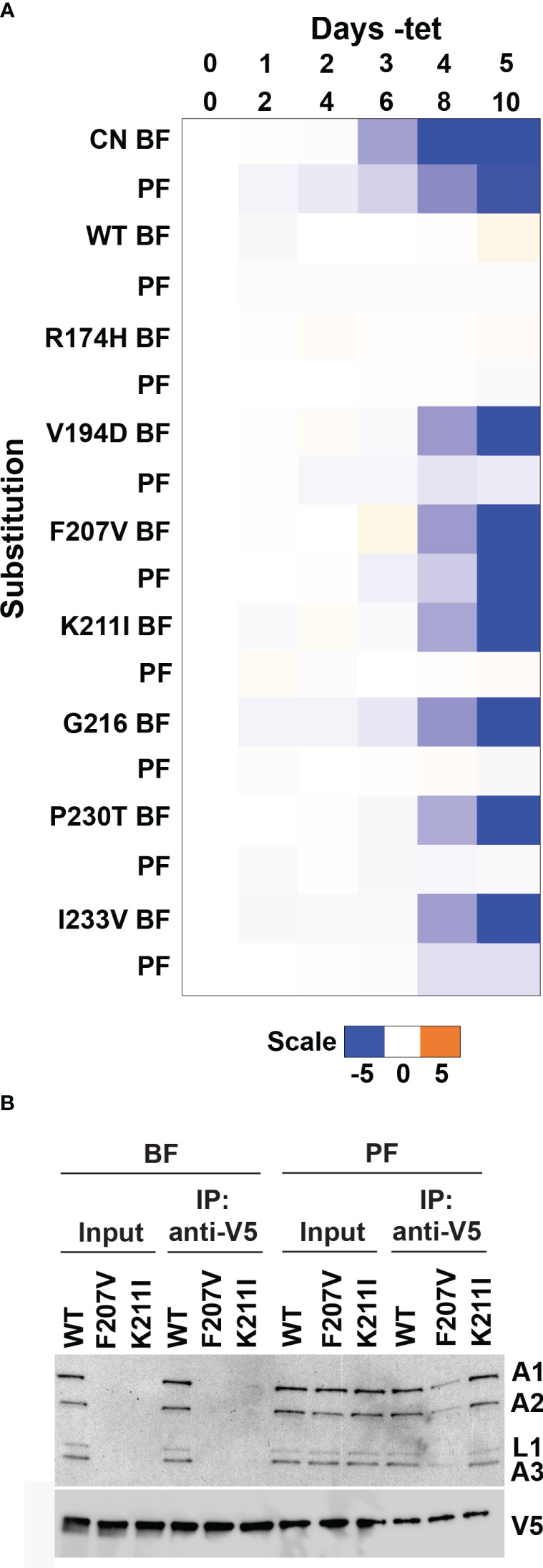
Life-cycle stage differences in fitness of cells containing LOF substitutions in B4. Alleles encoding single amino acid substitutions at selected residues (resulting in both LOF and no phenotype) identified by mutational scanning in BF cells were recreated by site-directed mutagenesis and transfected into the PF B4 CN cell line (McDermott and Stuart, 2017) for growth phenotype and RECC analysis. **(A)** The log2 ratio of the effect on cumulative growth in absence versus presence of tet is indicated by the scale showing reduction in blue, increase in orange, and no effect on growth in white. **(B)** BF or PF cells that exclusively expressed V5-tagged WT or mutant B4 following two or four days of repression of the regulatable WT B4 allele respectively were used for anti-V5 tag immunoprecipitation of B4-bound protein complexes. Cleared input cell lysates (5%) and anti-V5 immunoprecipitates (10%) were analyzed by Western blotting. Blots were probed with monoclonal antibodies against RECC proteins KREPA1, KREPA2, KREL1 and KREPA3, and anti-V5 antibody.

Several RECC protein mutagenesis studies, including of B5-B8 that contain RNase III-like domains, have identified single amino acid substitutions with different consequences on BF vs. PF cell growth and RECC integrities (Carnes et al., 2011; McDermott et al., 2015a, b; McDermott and Stuart, 2017; McDermott et al., 2019; Carnes et al., 2022; Davidge et al., 2023). Therefore, we hypothesized that we might also observe life cycle stage differences in the growth of cells containing substitutions that were identified in our BF B4 deep mutational scan. To test this hypothesis, we transfected selected site-directed V5-tagged LOF mutant constructs into our parental PF B4 CN cell line (McDermott and Stuart, 2017), where expression of the WT or mutant alleles was confirmed by Western analysis that probed for the V5 tag (**Supplementary Figure 9**). Exclusive expression of several B4 variants with BF LOF substitutions in the RNase III-like domain residues did not affect PF cell growth (**Figure 7A** and **Supplementary Data**). This included K211I, which in contrast to BF (**Figure 6C**), did not affect total levels or co-immunoprecipitation of RECC components in PF. The exception was the F207V variant that caused strong growth defects in both BF and PF cells (**Figures 3B**, **7A**), reduced total levels of RECC components in BF, and reduced co-immunoprecipitation of RECC components in PF (**Figure 7B**) compared to WT B4. In conclusion, as hypothesized, our screen identified single amino acid substitutions in the B4 RNase III-like domain that have different consequences on BF vs. PF cell growth and RECC integrities.

## 4 Discussion

Functional annotation of evolutionarily divergent trypanosomatid proteins with limited sequence homology to other eukaryotic proteins can be advanced by mutagenesis combined with novel high-throughput functional analyses (McDermott et al., 2015a; Carnes et al., 2022; Davidge et al., 2023). Here we describe the development of a deep mutational scanning approach for *T. brucei*, which makes use of tet-conditional expression of a wild-type gene of interest and constitutive expression of a library of mutant variants (McDermott et al., 2015a; McDermott and Stuart, 2017), a high-throughput sequencing readout for cell growth upon exclusive expression of mutant variants (Wei and Li, 2023), and freely available computational tools to estimate variant fitness (Faure et al., 2020). The method has improved upon our previous random mutagenesis and complementation approach (McDermott et al., 2015a; Carnes et al., 2022; Davidge et al., 2023) by increasing the number of substitutions that can be phenotypically screened by two orders of magnitude. This was enabled by both the high-throughput sequencing readout, and the generation of larger variant libraries in BF *T. brucei* via TMP/ddDHFR-induced I-SceI meganuclease stabilization and cleavage at the mutant library target locus. Guide RNA-mediated Cas cleavage at a target locus could also potentially be used in place of I-SceI target site cleavage (Liu et al., 2018; Zhang et al., 2020). Our approach uses *T. brucei* fitness and growth for functional selection and as a surrogate measure of protein function. Importantly, we expect that this method will also further contribute to our ability to screen for substitutions associated with phenotypes beyond straightforward effects on cell fitness. For example, these could include screening for substitutions that impact target protein stabilities via tagging with fluorescent reporters, or interactions with binding partners via display methods (Wei and Li, 2023). Furthermore, since homologous proteins of other important trypanosomatid parasites, including *T*. *cruzi* and *Leishmania*, can sometimes complement for loss of protein function in *T. brucei* (Rusconi et al., 2005; Carnes et al., 2012; Cestari et al., 2016), the method may be extended to the high-throughput study of certain *T*. *cruzi* and *Leishmania* proteins. We anticipate that this could be particularly important for assessment and prediction of mutations in trypanosomatid drug targets or prodrug activators that lead to altered drug efficacies or drug resistance (Hall et al., 2011; Collett et al., 2019; Altmann et al., 2022).

Here we used our deep mutational scanning method to query the functions of 83 residues in the B4 RNase III-like domain in BF *T. brucei*. The screen covered all 83 residues and analyzed the fitness effects of multiple substitutions at most positions. We identified 102 single amino acid substitutions at 61 of the 83 mutated sites that resulted in loss of B4 function in BF *T. brucei.* The results of the screen were validated using independently generated clonal BF B4 CN cell lines that constitutively expressed B4 variants with selected single substitutions. All these substitutions recapitulated the growth phenotypes observed in the deep mutational scanning screen. The results extend those of previous *T. brucei* B4 studies that analyzed the effects of site-directed substitutions in the RNase III-like domain (Carnes et al., 2012; McDermott and Stuart, 2017). The prior studies showed that a relatively conservative change in a conserved glycine residue (G163V) had a deleterious effect on cell growth and RECC integrity and protein abundances (McDermott and Stuart, 2017). However, several substitutions in other highly conserved residues, including at E164 in the RNase III-like domain, which is required for catalysis in other RNase III enzymes (Meng and Nicholson, 2008; Nicholson, 2014), did not prevent function or impact RECCs (Carnes et al., 2012; McDermott and Stuart, 2017). We previously interpreted this data as showing that the B4 RNase III-like domain does not have RNase III catalytic activity but is essential for maintaining RECC integrity, perhaps via an ability to interact with other RECC proteins, including via heterodimerization with other RNase III and RNase III-like domains in RECCs (McDermott and Stuart, 2017; McDermott et al., 2019). The multiple detrimental substitutions identified in this study and their position in the predicted B4 structure are consistent with this interpretation, particularly K211I, that maps to a region in proximity to other RECC proteins including N and B RNase III domain proteins, and severely impacts RECC integrity and protein abundances in BF cells.

Whether B4 is important for binding RNA substrates in addition to other RECC proteins is currently unknown and has not been tested directly. Our B4 structural model based on Rnt1p includes a bound double-stranded (ds)RNA, which we hypothesize could be a proxy for a gRNA-mRNA duplex e.g., in the anchor region, or following editing directed by the guiding region of the gRNA. Indeed, recent structural studies revealed that anchor and guiding region gRNA-mRNA duplexes protrude out from bound RNA editing substrate complexes (RESCs), presumably making them available for binding by RECC proteins (Liu et al., 2023). Our model predicts that B4 directly contacts bound dsRNA, specifically via its matrin-type zinc finger. This is also observed in models of other RNase III and RNase III-like domain-containing RECC proteins, including of their heterodimers. In these models, a zinc finger from each protein in the heterodimer is in contact with opposite sides of the dsRNA (Carnes et al., 2022), playing the role of dsRBD domains observed in other classical RNase III proteins (Meng and Nicholson, 2008; Nicholson, 2014). We previously showed that B4 likely interacts with N1, N2, and N3 (McDermott et al., 2016; McDermott and Stuart, 2017). It is therefore possible that a B4:N1/2/3 heterodimer binds gRNA-mRNA substrate during or following the catalytic steps of editing. Our previous protein-protein crosslinking studies are somewhat consistent with this since they did not identify crosslinks to other proteins in the vicinity of the modeled dsRNA (McDermott et al., 2016).

The current work adds to several RECC protein mutagenesis studies (McDermott et al., 2015a, b; Carnes et al., 2022; Davidge et al., 2023) that have identified single amino acid substitutions resulting in different consequences on BF vs. PF cell fitness and RECCs, despite RECCs apparently having the same protein compositions in BF and PF (Carnes et al., 2011). In all cases, BF RECCs and RECC components appear to generally be more sensitive to perturbation such as protein knockdown or mutation than those in PF. We previously interpreted this phenomenon as indicating numerous structural and functional differences between BF and PF RECCs, which could occur in response to changes in external and internal stimuli, e.g., temperature, surface protein expression, metabolites, and other soluble factors, that occur during the trypanosome life cycle. Interestingly, several B4 substitutions identified here, including K211I, have BF but not PF growth defects. Multiple of these are in a loop (N215-P234; **Supplementary Table 3**) that is predicted to be unstructured or disordered based on AlphaFold pLDDT and Fuzdrop pDP scores (Steinegger and Soding, 2017; Wheeler, 2021; Hatos et al., 2022). K211 is also close to this loop and the sites of these other substitutions. Thus, the life cycle stage specificity in the phenotypes of these substitutions might be explained by the ability of this region to fold and adopt alternative conformations depending upon stage differences in the extra- and intracellular environment. In contrast, substitutions that impact cell growth and RECC integrity in both BF and PF cells such as F207V may have broader impacts on B4 structure or stability. For example, in our predicted structure, F207 is in a position where B4 helices are potentially contacting each other, and we speculate that sequence disruption in this region may instead influence gross B4 structure regardless of life cycle stage.

Overall, this study demonstrates the utility of an unbiased deep mutational scanning approach to generate and assay many possible amino acid substitutions within a protein of interest on *T. brucei* cell growth. Our previous B4 site-directed mutagenesis studies were limited to just six total residues that were chosen based on their sequence conservation in B4 orthologs across kinetoplastid species (Carnes et al., 2012; McDermott and Stuart, 2017), and only substitutions at one of these sites resulted in detrimental phenotypic effects on cell growth and RECC integrity. Here, the ability to agnostically screen multiple substitutions at many residues allowed us to verify that there is no correlation between cell fitness phenotypes and residue conservation in the B4 RNase III-like domain, which demonstrates that sequence conservation cannot be used to predict B4 function and highlights the power of our deep mutational scanning method. We foresee that the approach could also be further improved, for example by using site saturation mutagenesis via gene synthesis or oligo pools instead of error-prone PCR to generate variants, which would increase the proportion of variant sequences with single substitutions and decrease the proportion of those with multiple substitutions. Variant barcoding (Starr et al., 2020; Kuiper et al., 2022; Wei and Li, 2023) would also remove the amplicon sequencing-based size constraint for the protein domain to be screened, thus enabling screening of larger protein domains and full-length proteins. This relatively straightforward mutagenesis approach can therefore be used to further our understanding of structure-function relationships in any essential trypanosome protein.

## Data availability statement

The datasets presented in this study can be found in online repositories. The names of the repository/repositories and accession number(s) can be found below: https://www.ncbi.nlm.nih.gov/sra/?term=PRJNA1071277, PRJNA1071277.

## Author contributions

SM: Conceptualization, Data curation, Formal analysis, Funding acquisition, Investigation, Methodology, Project administration, Supervision, Validation, Visualization, Writing – original draft, Writing – review & editing. VP: Investigation, Writing – review & editing. BO: Investigation, Writing – review & editing. JC: Conceptualization, Writing – review & editing. DS: Supervision, Writing – review & editing. KS: Funding acquisition, Project administration, Supervision, Writing – review & editing.
