# Supplementary figures and images for: Deep mutational scanning of the RNase III-like domain in Trypanosoma brucei RNA editing protein KREPB4
Source: Front Cell Infect Microbiol. 2024 Apr 8;14:1381155. doi: 10.3389/fcimb.2024.1381155 (PMC11033214; doi:10.3389/fcimb.2024.1381155)

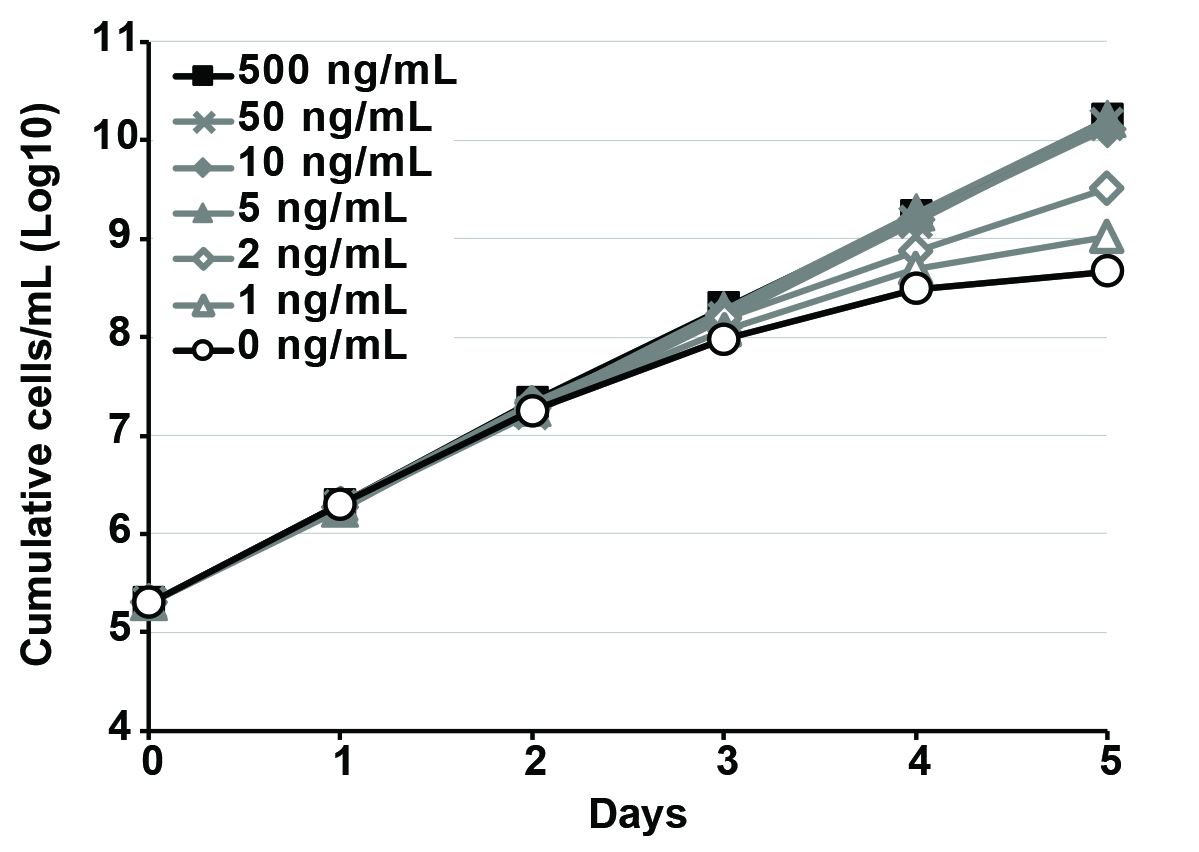

Supplement: Supplementary file 1 [file Image_1.tif]

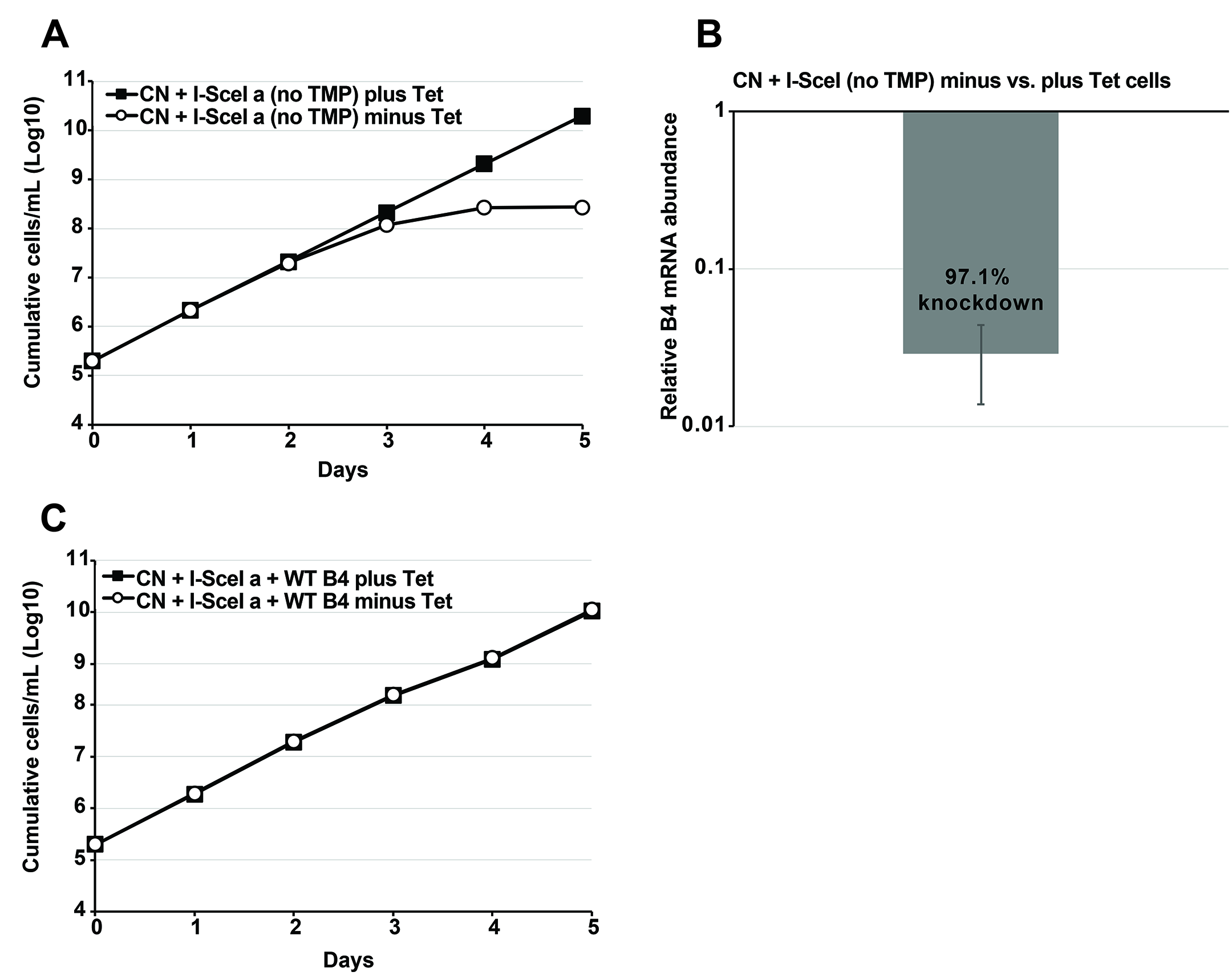

Supplement: Supplementary file 2 [file Image_2.tif]

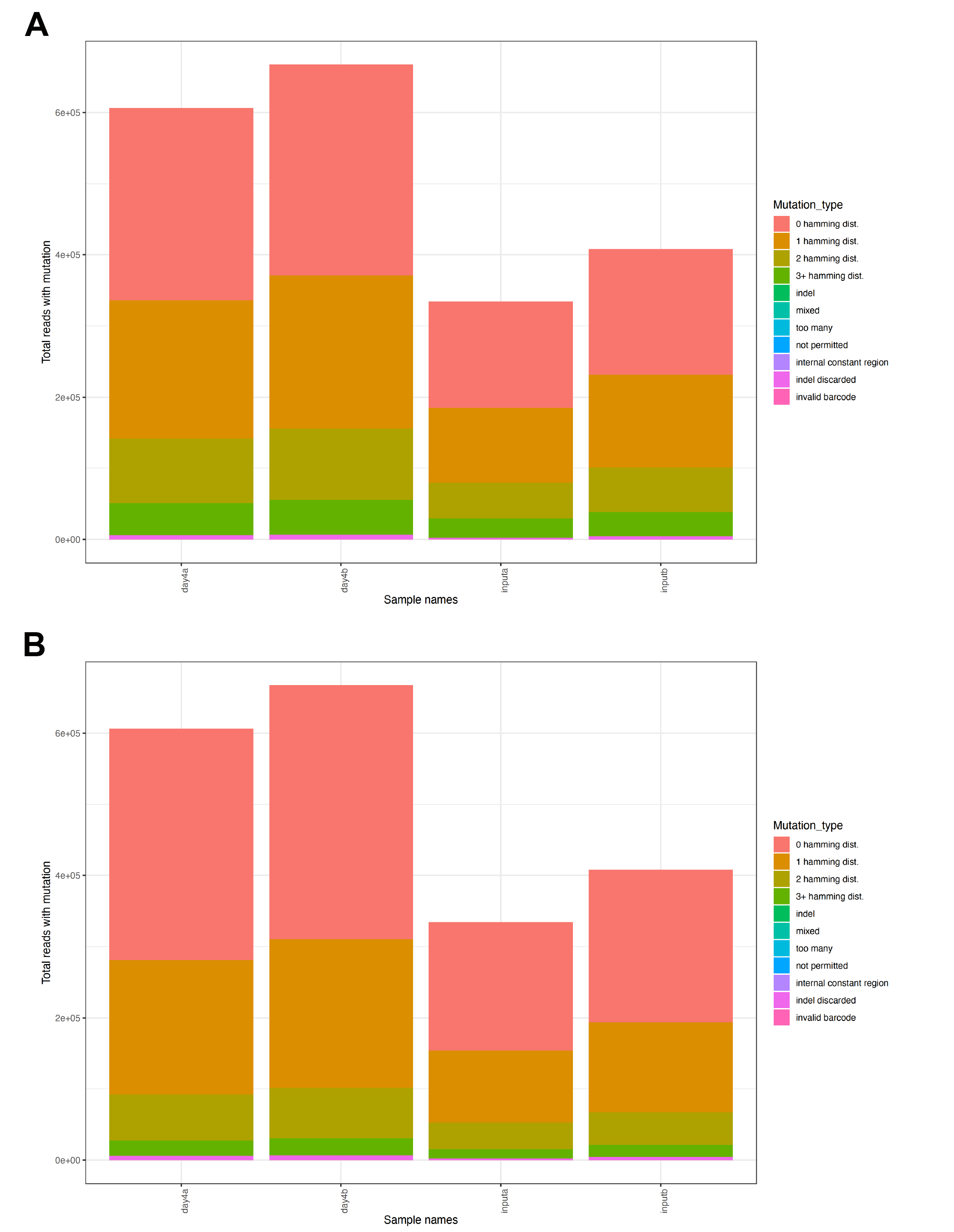

Supplement: Supplementary file 3 [file Image_3.tif]

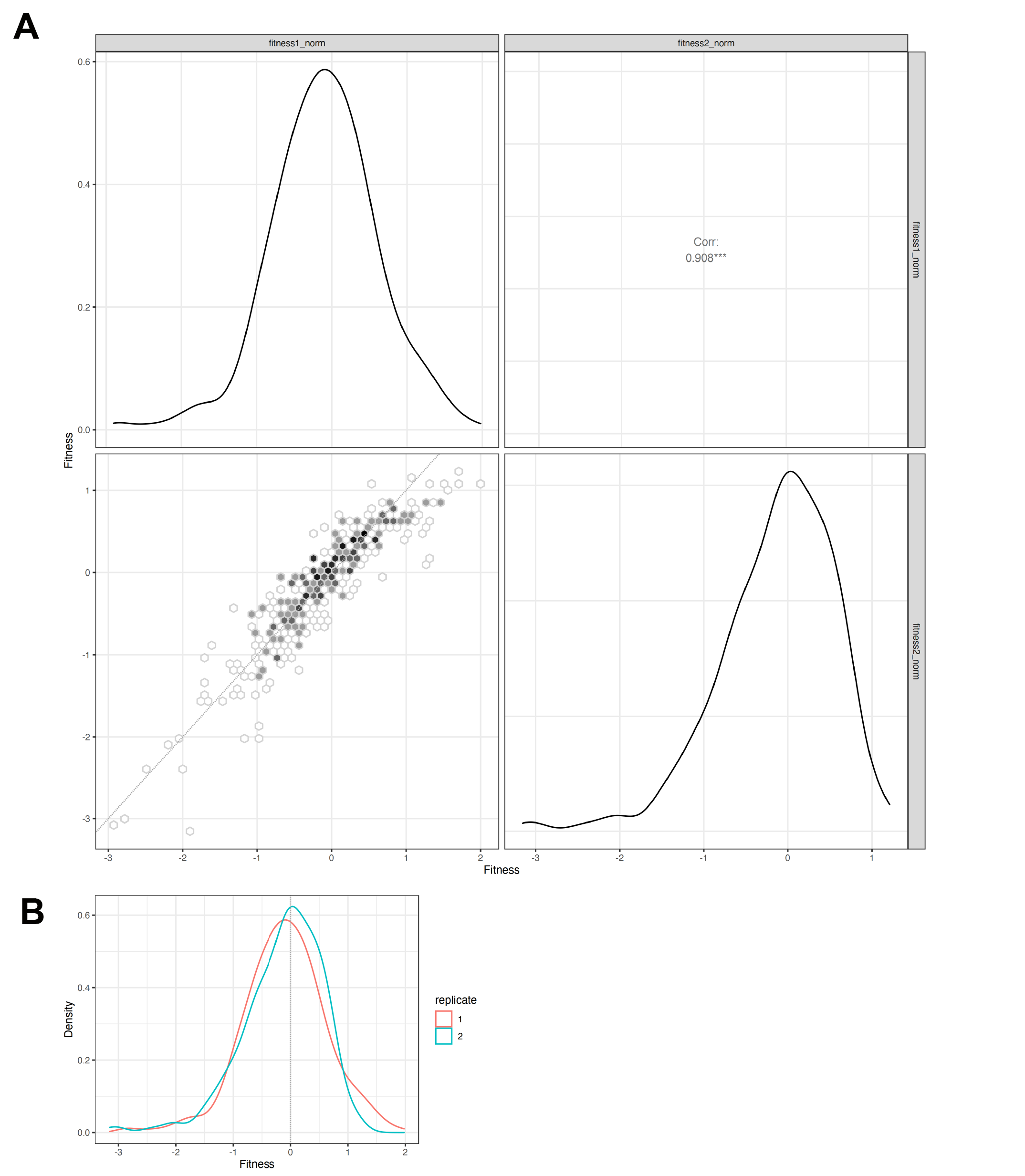

Supplement: Supplementary file 4 [file Image_4.tif]

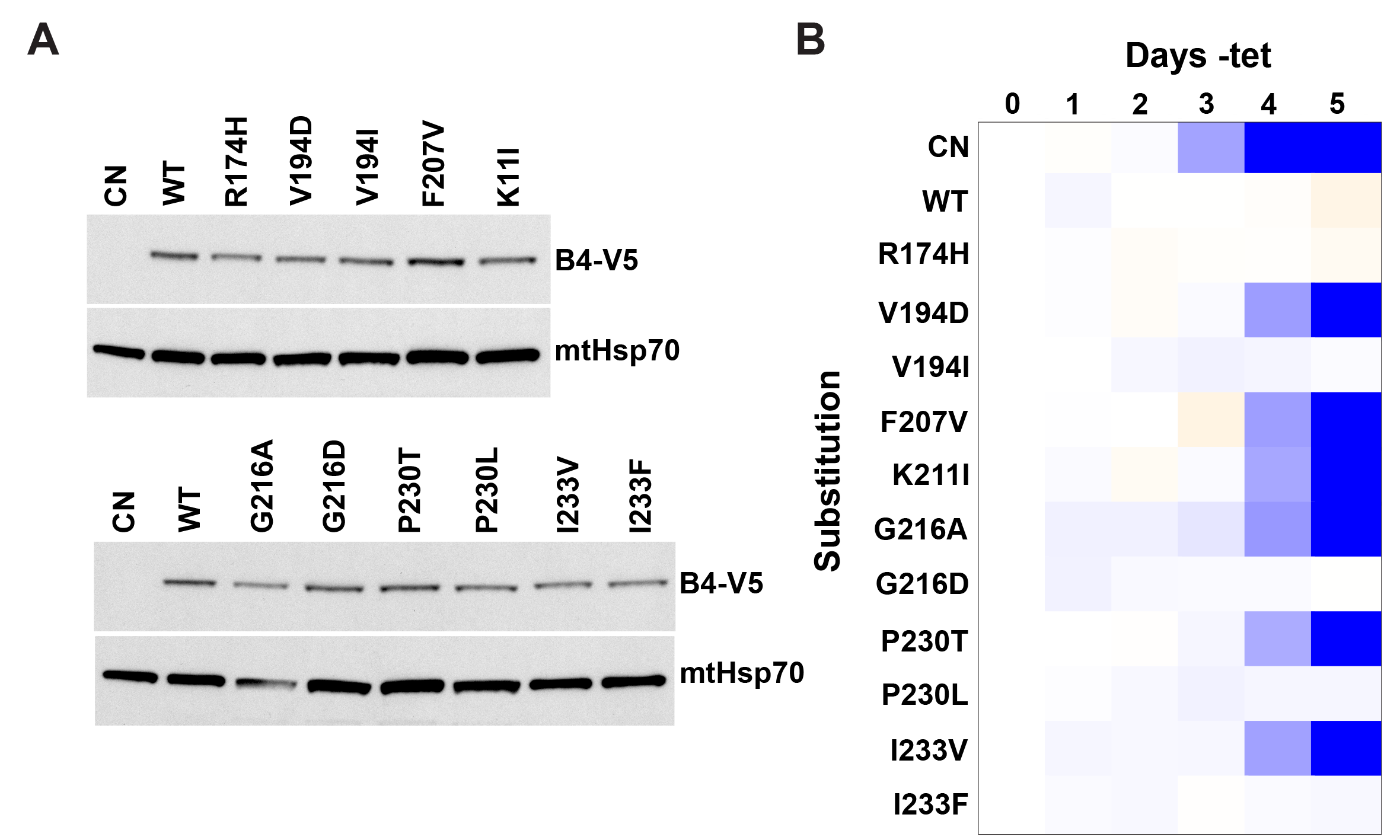

Supplement: Supplementary file 5 [file Image_5.tif]

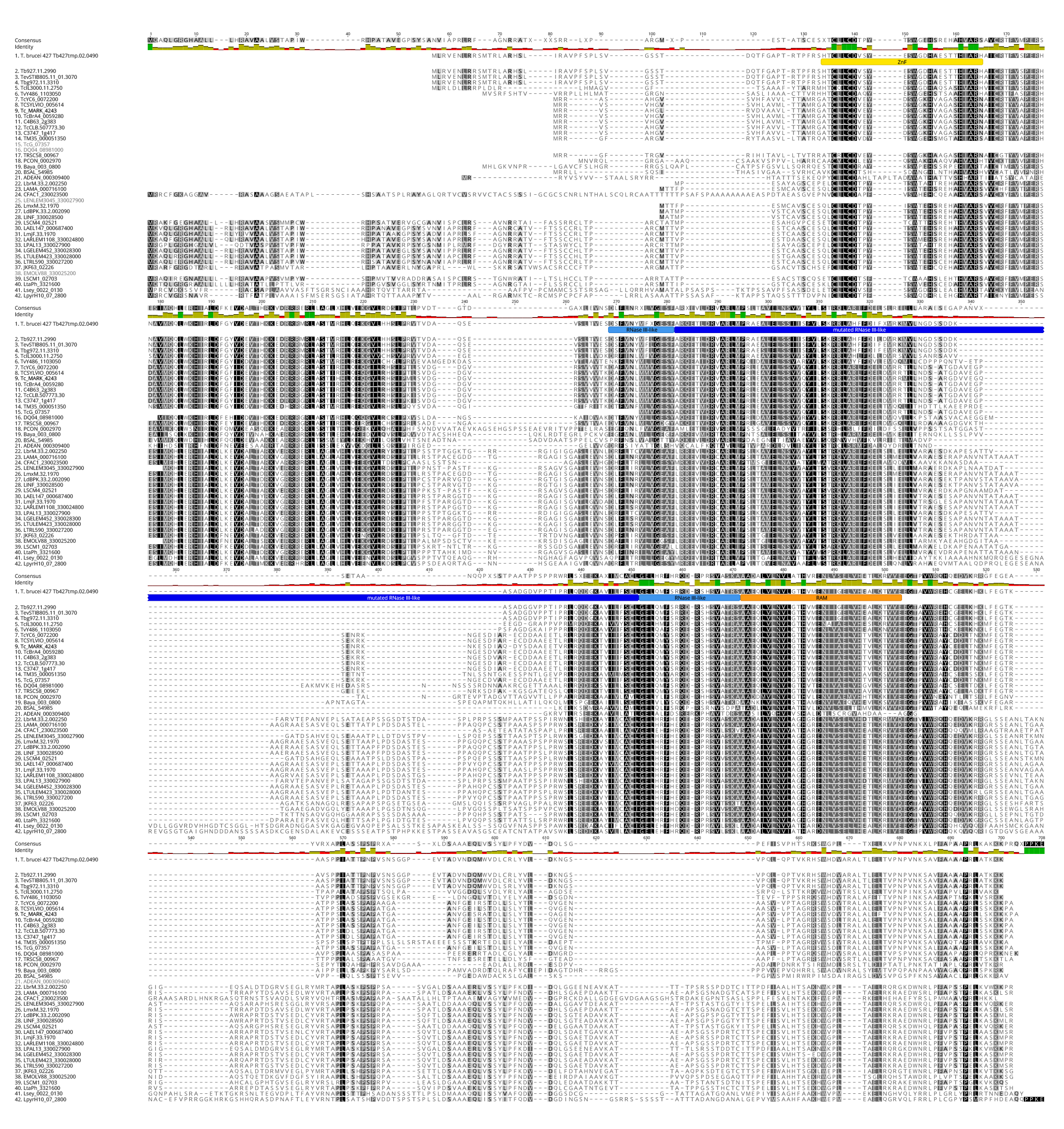

Supplement: Supplementary file 6 [file Image_6.tif]

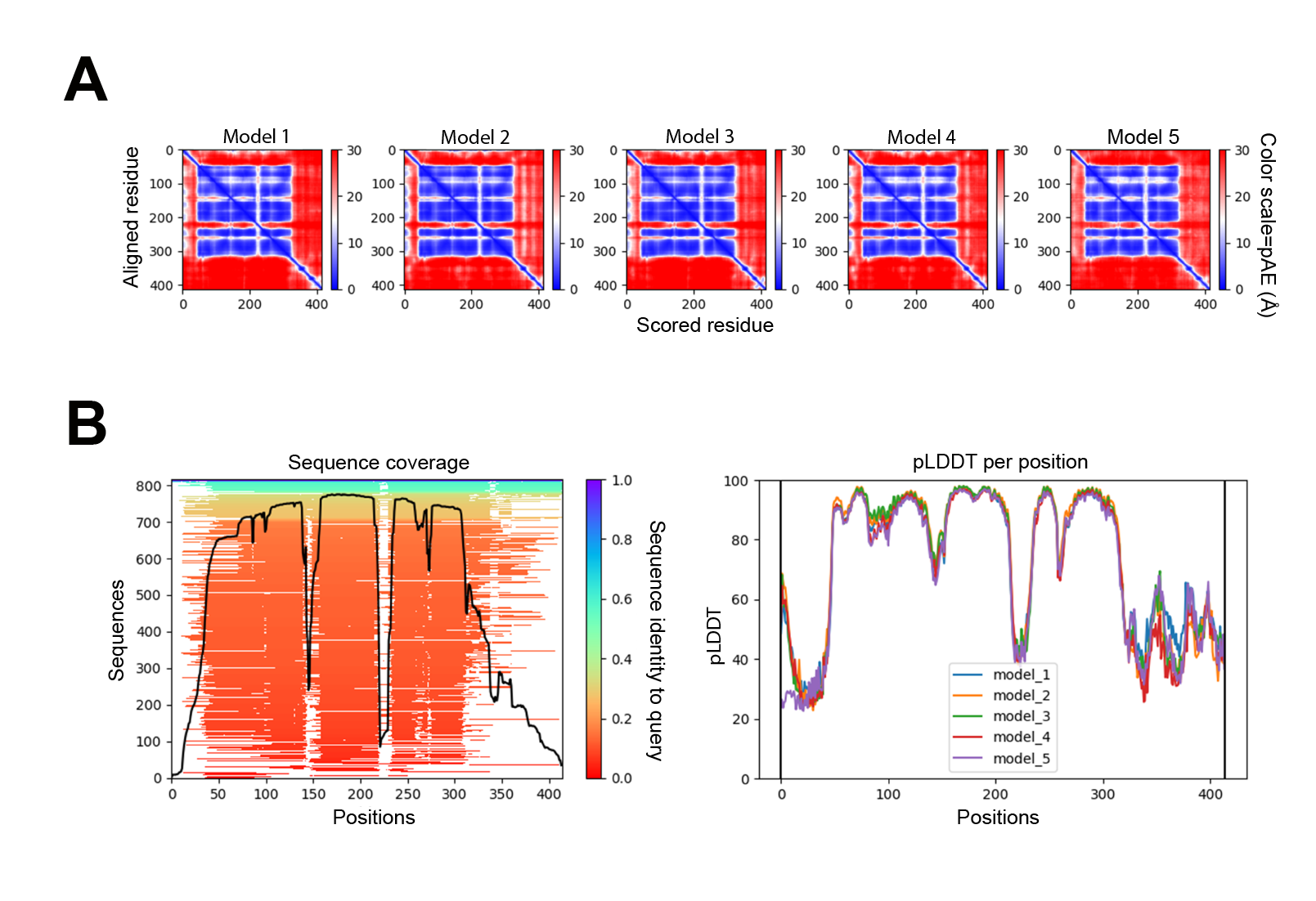

Supplement: Supplementary file 7 [file Image_7.tif]

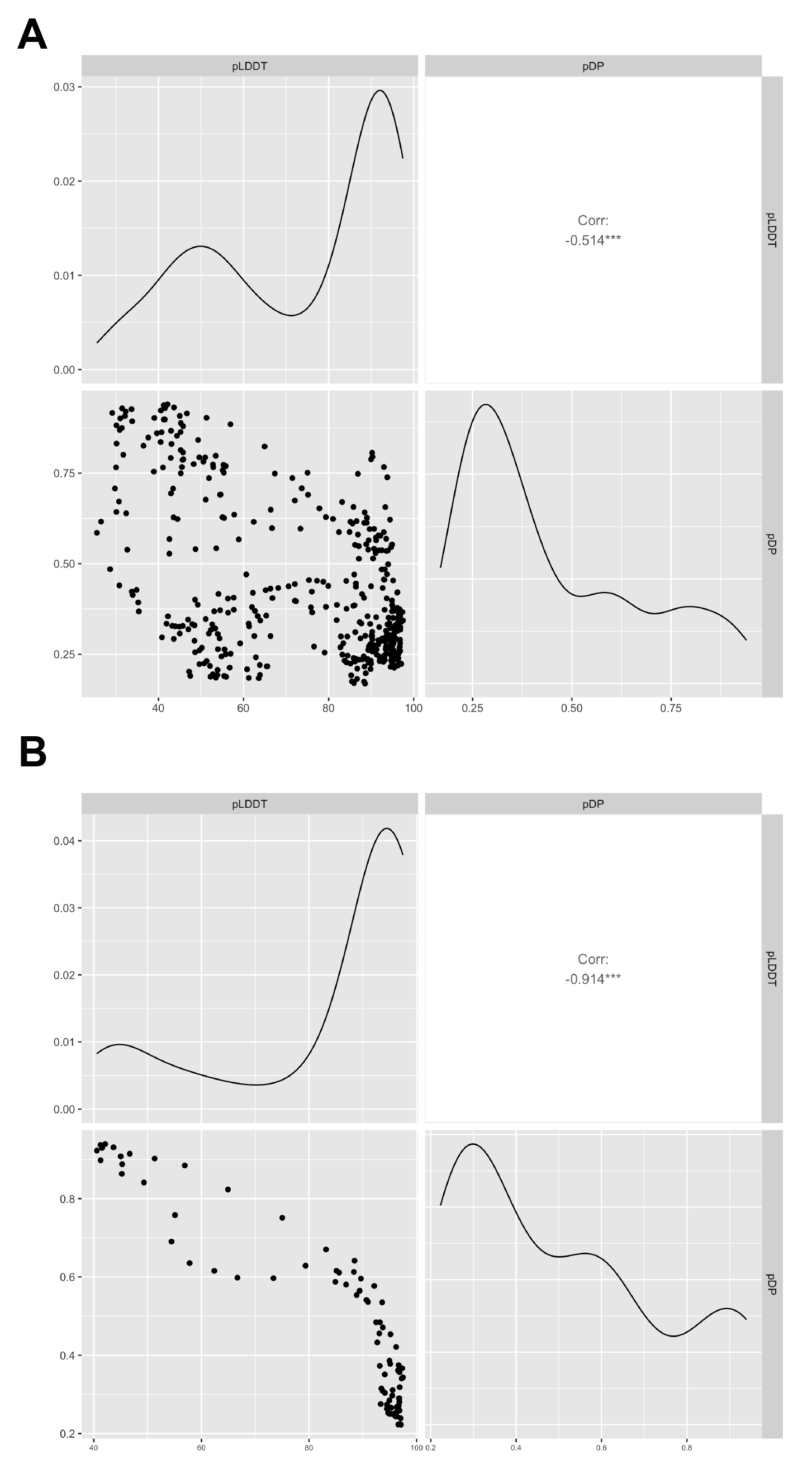

Supplement: Supplementary file 8 [file Image_8.tif]

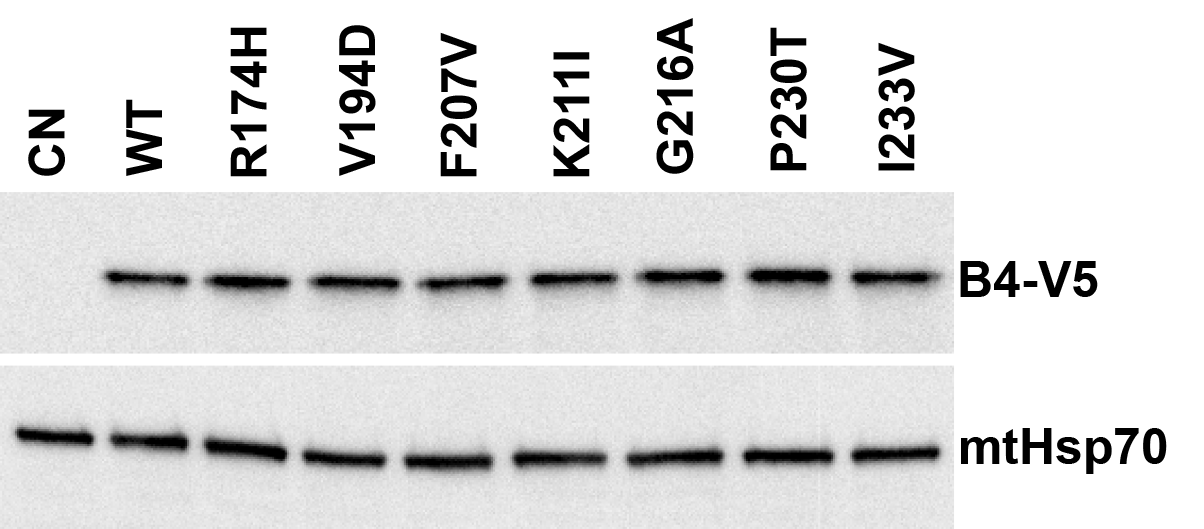

Supplement: Supplementary file 9 [file Image_9.tif]
