# Supplementary material for: Deep mutational scanning of the RNase III-like domain in Trypanosoma brucei RNA editing protein KREPB4
Source: Front Cell Infect Microbiol. 2024 Apr 8;14:1381155. doi: 10.3389/fcimb.2024.1381155 (PMC11033214; doi:10.3389/fcimb.2024.1381155)
Supplement: Supplementary file 10 [file DataSheet_1.docx]

Supplementary Material

# Supplementary Figures and Tables

## Supplementary Figure 1

## Supplementary Figure 2

## Supplementary Figure 3

## Supplementary Figure 4

## Supplementary Figure 5

## Supplementary Figure 6

## Supplementary Figure 7

## Supplementary Figure 8

## Supplementary Figure 9

## Supplementary Figure Legends

**Supplementary Figure 1. Minimum tet concentration required for survival of parental BF CN cells.** Growth of BF B4 CN cells at a range of tetracycline concentrations. The minimum concentration required for robust cell growth was 5 ng/mL.

**Supplementary Figure 2. Constitutive expression of a WT B4 allele rescues growth of BF B4 CN + I-SceI cells in the absence of tetracycline. (A)** BF B4 CN cells containing the I-SceI construct (grown in the absence of TMP) respond to tet-withdrawal with the same kinetics as the parental BF B4 CN cells (McDermott and Stuart, 2017), i.e., growth inhibition apparent 3 days following tet withdrawal. **(B)** Real-time PCR analysis shows loss of B4 mRNA expression following 2 days of tet-withdrawal from BF B4 CN cell lines that contain the I-SceI construct (grown in the absence of TMP). The relative change in B4 mRNA abundance in minus vs. plus tet cells was determined by using telomerase reverse transcriptase (TERT) mRNA as an internal control. Data are shown as means ± SEM from three independent cell lines (CN + SceI a, b, or c in Table 1). **(C)** The WT allele replaced the I-SceI-ddDHFR/I-SceI site construct in the tubulin locus as shown in Figure 2. Growth curves in (A) and (B) are shown for the ‘a’ cell line but are representative of three independent lines tested for both the BF B4 CN + I-SceI, or B4 CN + I-SceI + WT cells.

**Supplementary Figure 3. Nucleotide and amino acid variant statistics per input (plus tet) and selected (day 4; minus tet) replicate sample.** Total number of read counts with **(A)** nucleotide or **(B)** amino acid variant characteristics as indicated. Reads in each sample were retained or discarded for analysis in DiMSum (Faure et al., 2020) according to the following criteria: '0 hamming dist.' (retained: wild-type sequence); '1 hamming dist.' (retained: 1 nucleotide or amino acid substitution from wild-type sequence); '2 hamming dist.' (retained: 2 nucleotide or amino acid substitutions from wild-type sequence); '3+ hamming dist.' (retained: >3 nucleotide or amino acid substitutions from wild-type sequence); 'indel' (not applicable: insertion or deletion variant); 'mixed' (not applicable: nonsynonymous variants have synonymous substitutions in other codons); 'too many' (>9 substitutions discarded: too many amino acid substitutions); 'not permitted' (not applicable: nucleotide or amino acid substitution not permitted); 'internal constant region' (not applicable: nucleotide or amino acid substitution within internal constant sequence); 'indel discarded' (discarded: insertion or deletion variant); 'invalid barcode' (not applicable: reads represent invalid barcode sequences).

**Supplementary Figure 4. Fitness distributions and correlations for replicate selections. (A)** Scatterplot matrix depicting correlations between fitness estimates from each replicate selection following wild-type normalization. Upper-right matrix cell shows Pearson correlation coefficient. Lower-left matrix cell shows scatterplot heatmaps of fitness score per variant per replicate). Upper-left and lower-right matrix cells indicate fitness densities for each replicate. **(B)** Overlap of replicate fitness distributions in (A). Similar distributions indicate a lack of systematic errors between replicates (Faure et al., 2020). The fitness of wild-type sequences is indicated by the vertical dashed line (fitness score = 0).

**Supplementary Figure 5. Validation of growth phenotypes observed in mutational scanning screen.** Alleles encoding single amino acid substitutions at selected residues (resulting in both LOF and no phenotype) identified by mutational scanning were recreated by site-directed mutagenesis and transfected into CN cell lines for growth phenotype validation. **(A)** Western blot analysis with anti-V5 tag monoclonal antibody showing constitutive expression of V5-tagged mutant or WT B4 proteins from the β-tubulin locus in BF B4 CN cells (equivalent of 2 x 10^6^ cells/lane) in the absence of tet. Loading was assessed by probing the same blot with an anti-mitochondrial (mt)Hsp70 monoclonal antibody (Allen et al., 1998). **(B)** The log2 ratio of the effect on cumulative growth in absence versus presence of tet is indicated by the scale showing reduction in blue, increase in orange, and no effect on growth in white.

**Supplementary Figure 6. Clustal Omega alignment of *T. brucei* B4 with 41 orthologs from related kinetoplastid species.** Mutated region of RNase III-like domain underlined with dark blue. Other regions are underlined as follows: full RNase III-like domain boundaries with light blue, ZnF with yellow, and RAM with orange. Species are listed in descending order of percentage sequence similarity with *T. brucei* Lister 427 as follows: 1, *T. brucei* Lister 427; 2, *T. brucei* TREU927; 3, *T. evansi* strain STIB 805; 4, *T. brucei gambiense* DAL972; 5, *T. congolense* IL3000; 6, *T. vivax* Y486; 7, *T. cruzi* Y C6; 8, *T. cruzi* Sylvio X10; 9, *T*. *cruzi marinkellei* strain B7; 10, *T. cruzi* Brazil A4; 11, *T. cruzi* Dm28c; 12, *T. cruzi* CL Brener Non-Esmeraldo-like; 13, *T. cruzi* TCC; 14, *T. theileri* Edinburgh; 15, *T. cruzi* strain G; 16, *T. grayi* ANR4; 17, *T. rangeli* SC58; 18, *Paratrypanosoma confusum* CUL13; 19, *Blechomonas ayalai* B08-376; 20, *Bodo saltans* strain Lake Konstanz; 21, *Angomonas deanei* strain Cavalho ATCC PRA-265; 22, *Leishmania braziliensis* MHOM/BR/75/M2904; 23, *L. amazonensis* MHOM/BR/71973/M2269; 24, *Crithidia fasciculata* strain Cf-Cl; 25, *L. enriettii* strain LEM3045; 26, *L. mexicana* MHOM/GT/2001/U1103; 27, *L. donovani* strain LV9; 28, *L. infantum* JPCM5; 29, *L. orientalis* MHOM/TH/2014/LSCM4; 30, *L. aethiopica* L147; 31, *L. major* strain Friedlin; 32, *L. arabica* strain LEM1108; 33, *L. panamensis* MHOM/COL/81/L13; 34, L. gerbilli strain LEM452; 35, *L. turanica* strain LEM423; 36*, L. tropica* L590; 37, *Porcisia hertigi* MCOE/PA/1965/C119; 38, *Endotrypanum monterogeii* strain LV88; 39, *L. martiniquensis* MHOM/TH/2012/LSCM1; 40, *L. tarentolae* Parrot Tar II; 41, *Leptomonas seymouri* ATCC 30220; 42, *Leptomonas pyrrhocoris* H10.

**Supplementary Figure 7. Modeling of B4 using AlphaFold2 with modifications for analyzing proteins from organisms within the Discoba clade. (A)** Predicted aligned error (pAE) for five B4 models. pAE is a per residue pair distance score (in Å) with low values showing lower error. **(B)** Sequence coverage and predicted per residue local distance difference test (pLDDT) scores for the five B4 models. Higher values indicate higher confidence in the model. pLDDT > 90 = very high confidence; 90 > pLDDT > 70 = high confidence; 70 > pLDDT > 50 = low confidence; pLDDT < 50 = very low confidence.

**Supplementary Figure 8. AlphaFold2 model confidence correlates with protein disorder.** Scatterplot matrices depicting correlations between predicted per residue local distance difference test (pLDDT) scores from our AlphaFold2 structure model, and residue-based droplet-promoting probabilities (pDPs), in **(A)** across the whole protein and in **(B)** across the randomly mutated RNase III-like region. pDP serves as a proxy for disorder (Hatos et al., 2022). Upper-right and lower-left matrix cells show Pearson correlation coefficient and scatterplots respectively. Lower-right and upper-left matrix cells are density plots showing the distribution of values used in the analysis for pLDDT and pDP respectively.

**Supplementary Figure 9. Generation of PF CN cells containing exclusively expressed B4 alleles with selected substitutions.** Alleles encoding single amino acid substitutions at selected residues (resulting in both LOF and no phenotype) identified by mutational scanning in BF cells were recreated by site-directed mutagenesis and transfected into the PF B4 CN cell line (McDermott and Stuart, 2017). Western blot analyses with anti-V5 tag monoclonal antibody show constitutive expression of V5-tagged mutant or WT B4 proteins from the β-tubulin locus in PF B4 CN cells (equivalent of 2 x 10^6^ cells/lane) in the absence of tet. Loading was assessed by probing the same blot with an anti-mitochondrial (mt)Hsp70 monoclonal antibody (Allen et al., 1998).

## Supplementary Tables

**Supplementary Table 1.** Oligonucleotide sequences used in this study. Restriction sites are underlined.

| **Primer Description** | **Primer sequence** | **Reference if applicable** |
| --- | --- | --- |
| Forward primer KREPB4 RNase III-like domain mutagenesis | TTCCTGGGCGAAAGCTTT | This study |
| Reverse primer KREPB4 RNase III-like domain mutagenesis | GAGAACATTTGCAACTCCCC | This study |
| Forward primer BamHI-L4 3’UTR | GATCGGATCCGCGTTAAGAGAGGAGAAAACC | This study |
| Reverse primer L4 3’UTR-I-SceI site | CATTACCCTGTTATCCCTAGAGCAACGAATATACGTACAA | This study |
| Forward primer I-SceI site-Aldolase 5’UTR | CTAGGGATAACAGGGTAATGTGCTCAAGCTGTGTAGCG | This study |
| Reverse primer Aldolase 5’UTR-SpeI | GATCACTAGTGGTAAGCTTCGTTGCAGTTGA | This study |
| Forward primer amplification L4 3’UTR, I-SceI site, aldolase 5’UTR cassettte | GATCGGATCCGCGTTAAGAGAG | This study |
| Reverse primer amplification L4 3’UTR, I-SceI site, aldolase 5’UTR cassettte | GATCACTAGTGGTAAGCTTCGTTG | This study |
| Forward primer BglII-nourseothricin cassette | GGATAGATCTATGACCACTCTTGACGAC | This study |
| Reverse primer nourseothricin cassette-XbaI | CCATTCTAGAGGGGCAGGGCATGCT | This study |
| Forward primer SpeI-nourseothricin-HSVTK cassette | GATCACTAGTATGACCACTCTTGACGAC | This study |
| Reverse primer nourseothricin-HSVTK cassette-Bsu36I | GATCCCTGAGGTCAGTTAGCCTCCCCCAT | This study |
| Forward primer HindIII-I-SceI-ddDHFR | GATCAAGCTTATGCCAAAGAAGAAGCGA | This study |
| Reverse primer I-SceI-ddDHFR-BamHI | GATCGGATCCCTAGCATGCTCGCCGCTC | This study |
| Forward primer KREPB4 ORF qPCR | TCACCACAGTGTTCACGCCATACT | (McDermott and Stuart, 2017) |
| Reverse primer KREPB4 ORF qPCR | ATGTGCTTGGCACACACGTAATGG | (McDermott and Stuart, 2017) |
| Forward primer TERT qPCR | GAGCGTGTGACTTCCGAAGG | (Brenndorfer and Boshart, 2010) |
| Forward primer TERT qPCR | AGGAACTGTCACGGAGTTTGC | (Brenndorfer and Boshart, 2010) |
| P5-TAGATCGC-F1 | AATGATACGGCGACCACCGAGATCTACACTAGATCGCTCGTCGGCAGCGTC | (Carnes et al., 2017) |
| P5-CTCTCTAT-F2 | AATGATACGGCGACCACCGAGATCTACACCTCTCTATTCGTCGGCAGCGTC | (Carnes et al., 2017) |
| P5-TATCCTCT-F3 | AATGATACGGCGACCACCGAGATCTACACTATCCTCTTCGTCGGCAGCGTC | (Carnes et al., 2017) |
| P5-AGAGTAGA-F4 | AATGATACGGCGACCACCGAGATCTACACAGAGTAGATCGTCGGCAGCGTC | (Carnes et al., 2017) |
| P5-GTAAGGAG-F5 | AATGATACGGCGACCACCGAGATCTACACGTAAGGAGTCGTCGGCAGCGTC | (Carnes et al., 2017) |
| P5-ACTGCATA-F6 | AATGATACGGCGACCACCGAGATCTACACACTGCATATCGTCGGCAGCGTC | (Carnes et al., 2017) |
| P5-AAGGAGTA-F7 | AATGATACGGCGACCACCGAGATCTACACAAGGAGTATCGTCGGCAGCGTC | (Carnes et al., 2017) |
| P5-CTAAGCCT-F8 | AATGATACGGCGACCACCGAGATCTACACCTAAGCCTTCGTCGGCAGCGTC | (Carnes et al., 2017) |
| P7-TCGCCTTA-R1 | CAAGCAGAAGACGGCATACGAGATTCGCCTTAGTCTCGTGGGCTCGG | (Carnes et al., 2017) |
| P7-CTAGTACG-R2 | CAAGCAGAAGACGGCATACGAGATCTAGTACGGTCTCGTGGGCTCGG | (Carnes et al., 2017) |
| P7-TTCTGCCT-R3 | CAAGCAGAAGACGGCATACGAGATTTCTGCCTGTCTCGTGGGCTCGG | (Carnes et al., 2017) |
| P7-GCTCAGGA-R4 | CAAGCAGAAGACGGCATACGAGATGCTCAGGAGTCTCGTGGGCTCGG | (Carnes et al., 2017) |
| P7-AGGAGTCC-R5 | CAAGCAGAAGACGGCATACGAGATAGGAGTCCGTCTCGTGGGCTCGG | (Carnes et al., 2017) |
| P7-CATGCCTA-R6 | CAAGCAGAAGACGGCATACGAGATCATGCCTAGTCTCGTGGGCTCGG | (Carnes et al., 2017) |
| P7-GTAGAGAG-R7 | CAAGCAGAAGACGGCATACGAGATGTAGAGAGGTCTCGTGGGCTCGG | (Carnes et al., 2017) |
| P7-CCTCTCTG-R8 | CAAGCAGAAGACGGCATACGAGATCCTCTCTGGTCTCGTGGGCTCGG | (Carnes et al., 2017) |
| Forward primer KREPB4 RNase III-like MiSeq adaptor | TCGTCGGCAGCGTCAGATGTGTATAAGAGACAGTTCCTGGGCGAAAGCTTT | This study |
| Reverse primer KREPB4 RNase III-like MiSeq adaptor | GTCTCGTGGGCTCGGAGATGTGTATAAGAGACAGGAGAACATTTGCAACTCCCC | This study |
| Reverse primer attB2-V5 | GAGAACATTTGCAACTCCCC | This study |

**Supplementary Table 2.** Counts of ORFs with different numbers of amino acid substitutions across all samples.

| **Number (#) of substitutions** | **All ORFs with # substitutions** | **Total ORFs per # substitutions** | **Unique ORFs with # substitutions** | **Total unique ORFs per # substitutions** |
| --- | --- | --- | --- | --- |
| 0 | 3303 | 3303 | 3303 | 3303 |
| 1 | 20,909 | 20,909 | 735 | 735 |
| 2 | 34,907 | 34,907 | 27,619 | 27,619 |
| 3 | 12,870 | 16,759 | 12,004 | 15,708 |
| 4 | 3,097 |  | 2,920 |  |
| 5 | 698 |  | 690 |  |
| 6 | 79 |  | 79 |  |
| 7 | 12 |  | 12 |  |
| 8 | 2 |  | 2 |  |
| 9 | 1 |  | 1 |  |

**Supplementary Table 3.** Single amino acid substitution variant fitness scores from combined replicate experiments and associated statistics. Table also reports 1) mean residue fitness score calculated using the scores for the different substitutions at each site; 2) per residue conservation score calculated for each amino acid as mean pairwise identity over all pairs per column in the full alignment of *T. brucei* B4 with 41 orthologs from a range of kinetoplastid species including *T. cruzi* and *L. major* (see Supplementary Figure 7 for full alignment); 3) per residue AlphaFold pLDDT and FusDrop pDP scores which are measures of confidence in the predicted position of the residue in the structural model and of potential disorder respectively. pLDDT and pDP are correlated (Supplementary Figure 9).

See excel file:

Supplementary Table 3.xlsx

**Supplementary Table 4.** Distances between crosslinked residues within B4 that are present in the structural models presented in Figure 5 and Supplementary Figure 10. From (McDermott et al., 2016).

| **B4 Residue 1** | **B4 Residue 2** | **B4 Residue 1-B4 Residue 2 C𝛂- C𝛂 distance (Å)** |
| --- | --- | --- |
| K95 | K110 | 25.53 |
| K95 | K111 | 25.86 |
| K222 | K211 | 19.93 |

**Supplementary Table 5.** Residues in B4 that are present in the structural models presented in Figure 5 and Supplementary Figure 10, that crosslinked with residues in other RECC proteins. From (McDermott et al., 2016). Interactions between B4 and other RECC proteins are generally found in regions that lack homology to other known domains, except for the A5 and A6 OB-folds and a ZnF in A2.

| **B4 Residue 1** | **RECC protein 2** | **RECC protein 2 Residue** | **RECC protein 2 domain** |
| --- | --- | --- | --- |
| K95 | N1 | K233 |  |
| K95 | B5 | K208 |  |
| K95 | A1 | K567 |  |
| K95 | A5 | K57 | A5 OB-fold |
| K110 | N2 | K181 |  |
| K110 | N3 | K135 |  |
| K110 | B6 | K336 |  |
| K110 | B10 | K348 |  |
| K110 | A1 | K567 |  |
| K129 | N3 | K485 |  |
| K129 | B6 | K336 |  |
| K129 | B5 | K343 |  |
| K129 | A2 | K246, K415, K447 | A2 ZnF2 (K246) |
| K129 | A6 | K149 | A6 OB-fold |
| K197 | A4 | K69 |  |
| K314 | A1 | K480 |  |
| K314 | A4 | K69 |  |
| K314 | A6 | K88 | A6 OB-fold |

# Supplementary Data

Supplementary B4 Rnt1p RNaseIII modeling parameters.docx (Data Sheet 2)

Supplementary B4 BF mutant growth curves.xlsx (Data Sheet 3)

Supplementary B4 PF mutant growth curves.xlsx (Data Sheet 4)

## Supplementary References

Allen, T.E., Heidmann, S., Reed, R., Myler, P.J., Goringer, H.U., Stuart, K.D., 1998. Association of guide RNA binding protein gBP21 with active RNA editing complexes in Trypanosoma brucei. Molecular and Cellular Biology 18, 6014-6022. doi:

Brenndorfer, M., Boshart, M., 2010. Selection of reference genes for mRNA quantification in Trypanosoma brucei. Molecular and Biochemical Parasitology 172, 52-55. doi: 10.1016/j.molbiopara.2010.03.007

Carnes, J., McDermott, S., Anupama, A., Oliver, B.G., Sather, D.N., Stuart, K., 2017. In vivo cleavage specificity of Trypanosoma brucei editosome endonucleases. Nucleic Acids Research 45, 4667-4686. doi: 10.1093/nar/gkx116

Faure, A.J., Schmiedel, J.M., Baeza-Centurion, P., Lehner, B., 2020. DiMSum: an error model and pipeline for analyzing deep mutational scanning data and diagnosing common experimental pathologies. Genome Biol 21, 207. doi: 10.1186/s13059-020-02091-3

Hatos, A., Tosatto, S.C.E., Vendruscolo, M., Fuxreiter, M., 2022. FuzDrop on AlphaFold: visualizing the sequence-dependent propensity of liquid-liquid phase separation and aggregation of proteins. Nucleic Acids Research 50, W337-W344. doi: 10.1093/nar/gkac386

McDermott, S.M., Luo, J., Carnes, J., Ranish, J.A., Stuart, K., 2016. The Architecture of Trypanosoma brucei editosomes. Proceedings of the National Academy of Sciences of the United States of America 113, E6476-E6485. doi: 10.1073/pnas.1610177113

McDermott, S.M., Stuart, K., 2017. The essential functions of KREPB4 are developmentally distinct and required for endonuclease association with editosomes. RNA 23, 1672–1684. doi: 10.1261/rna.062786.117
