# Supplementary material for: Deep mutational scanning of the RNase III-like domain in Trypanosoma brucei RNA editing protein KREPB4
Source: Front Cell Infect Microbiol. 2024 Apr 8;14:1381155. doi: 10.3389/fcimb.2024.1381155 (PMC11033214; doi:10.3389/fcimb.2024.1381155)
Supplement: Supplementary file 11 [file DataSheet_2.docx]

**Matchmaker modelling parameters**

PDB 5T16 (*S. cerevisiae* Rnt1p)

Matchmaker 5T16, chain A (#0) with B4_dd465_unrelaxed_model_1.pdb, chain A (#1), sequence alignment score = 145.5

with these parameters:

chain pairing: ss

Smith-Waterman using BLOSUM-62

ss fraction: 0.3

gap open (HH/SS/other) 18/18/6, extend 1

ss matrix: (O, S): -6 (H, O): -6 (H, H): 6 (S, S): 6 (H, S): -9 (O, O): 4

iteration cutoff: 2

RMSD between 68 pruned atom pairs is 0.997 angstroms; (across all 118 pairs: 11.214)
